# The critical role of matrix metalloproteinase 9-mediated microglial polarization in perioperative neurocognitive disorders of aged rats

**DOI:** 10.3389/fimmu.2025.1650254

**Published:** 2025-08-21

**Authors:** Xi Xin, Haonan Zhang, Chenyi Yang, Xinyi Wang, Lin Zhang, Ji Ma, Haiyun Wang

**Affiliations:** ^1^ The Third Central Clinical College of Tianjin Medical University, Tianjin, China; ^2^ Department of Anesthesiology, Tianjin University Central Hospital, Tianjin, China; ^3^ Tianjin Key Laboratory of Extracorporeal Life Support for Critical Diseases, Tianjin, China; ^4^ Artificial Cell Engineering Technology Research Center, Tianjin, China; ^5^ Tianjin Institute of Hepatobiliary Disease, Tianjin, China; ^6^ Nankai University, Tianjin, China; ^7^ Nankai University Affinity the Third Central Hospital, Tianjin, China; ^8^ Tianjin University, Tianjin, China

**Keywords:** neuroinflammation, matrix metalloproteinase 9, perioperative neurocognitive disorders, microglia, aged

## Abstract

**Background:**

Perioperative neurocognitive disorders (PND) is a significant clinical syndrome and neuroinflammation is an important pathological process. Matrix metalloproteinase 9 (MMP9) as a Zn2+-dependent matrix enzyme, not only maintains the integrity of the blood-brain barrier and synaptic plasticity, but also plays a key regulatory factor in peripheral and central nervous inflammation. This study aimed to investigate the effects of MMP9-mediated microglial polarization on surgery-induced neuroinflammation in aged rats and to provide novel targets for prevention and treatment of PND.

**Methods:**

This study utilized an intraperitoneal injection of SB-3CT, an MMP9 inhibitor, to impede the action of MMP9. Morris water maze and novel object recognition test were conducted to assess behavioral performances. Western blot was employed to examine hippocampal inflammatory factors. Immunofuorescence and flow cytometry were used to examine the transformation of microglia phenotype.

**Results:**

The findings demonstrated that surgical intervention induced significant impairment in learning and memory performance in aged rats, accompanied by elevated MMP9 expression, exacerbated hippocampal inflammation, and microglial polarization characterized by a predominant M1 phenotype. Administration of SB-3CT effectively reversed these pathological manifestations.

**Conclusion:**

The inhibition of MMP9 can enhance neurological function by modulating the polarization of microglia and alleviating neuroinflammation, which is a new approach for perioperative neuroprotection in high-risk PND patients.

## 1 Introduction

Perioperative neurocognitive disorders (PND) is a prevalent complication of major surgeries especial amongst geriatric, including preoperatively diagnosed cognitive dysfunction and acute and chronic cognitive dysfunction diagnosed from one day to one year after surgery (1). In non-cardiac surgery, the incidence of PND ranges from 8.9% to 46.1%, depending on the type of surgery (2), and progressively increases with age. Notably, the incidence of PND is particularly high in elderly patients with preoperative neurodegeneration. PND, associated with poor functional recovery, increases the overall postoperative morbidity and mortality by 10% to 20% and to increase the average annual cost by $164 billion (3). PND not only affects the ability and quality of life, but also is related to the degeneration of the central nervous system (CNS) (4–9). Therefore, it is imperative to implement effective measures to prevent PND, with the aim of enhancing patient quality of life and recovery, and alleviating the burden on families and society.

Although the pathogenesis of PND remains unclear, preclinical studies suggest that surgery triggers acute systemic inflammation and blood-brain barrier dysfunction followed by neuroinflammation and synaptic dysfunction, which appear to contribute to hippocampal-dependent cognitive deficits (10, 11). Neuroinflammation is characterized by reactive gliosis and production of soluble inflammatory factors in the CNS, with a sustained inflammatory response involving microglia and astrocytes. Microglia interact with almost all CNS components and peripheral-invading immune cells, when they carry out a large number of non-immune and immune tasks that are crucial for brain function (12). Microglia are de-branched after activation and promptly change their phenotype, which differentiate into M1-type and M2-type microglia, mediating neuroprotective or inevitable detrimental effects (13). The advent of single-cell technologies provided clear evidence that microglia in the living brain are more complex than simply M1 and M2 (14). Microglia have multidimensional activation states in CNS diseases and injuries, such that these cells can have beneficial or detrimental roles depending on the context. M1/M2 dichotomy is a simplified model used to describe the functional state of microglia (pro-inflammatory versus anti-inflammatory). When the CNS is exposed to low levels of neuroinflammation for a short duration, microglia play a protective role. Conversely, the chronic persistent neuroinflammation inhibits neuronal regeneration, contributing to the development of PND. It has been demonstrated that sustained induction of microglial polarization leads to myelin loss and astrocyte activation, resulting in PND in mice (15). Consequently, the proportion of microglial polarization has been shown to determine the development and regression of PND. However, the molecular pathway that drives the microglia phenotypic changes during postoperative cognitive impairment remains unclear.

Matrix metalloproteinase 9 (MMP9) is a Zn^2+^-dependent metallo-matrix protease, which hydrolyzes intercellular tight junctions of collagenase and also acts as a crucial inflammatory factor with regulatory and signaling roles. Intracranial injection of Aβ has been demonstrated to induce MMP9 expression and hippocampal damage associated with learning and memory deficits, which was attenuated by MMP9 inhibitors (16). Meanwhile, MMP9 derived peripheral neutrophils contribute to postoperative cognitive impairment in aged mice by increasing the permeability of the blood-brain barrier (17). MMP9 can increase intracranial inflammation levels by altering blood-brain barrier permeability and modulating inflammatory factors. Additionally, increased MMP9 levels in the brain indirectly aid tau aggregation, resulting in harmful side effects on crucial brain regions. Therefore, elevated MMP9 in both peripheral and CNS can lead to cognitive dysfunction.

An earlier study revealed that microglia-mediated MMP9 activation impairs the blood-brain barrier, exacerbates dopamine neurodegeneration, and accelerates the progression of CNS diseases (18). Furthermore, the impaired blood-brain barrier function caused by MMP9 expression will subsequently activate microglia, leading to increased MMP9 secretion through a positive feedback loop (19). Conversely, microglia depletion or the inhibition of MMP9 activity can reverse the cognitive impairment induced by glial activation (20). Furthermore, an increase in MMP9 was detected in the hippocampus of an animal model of tibial fracture, accompanied by blood-brain barrier disruption and cognitive decline (21). Therefore, regulation of MMP9 may be an effective way to accommodate neuroinflammation. However, the relationship between MMP9 and microglial polarization has not been elucidated, and further basic studies are needed to verify the nature of the association between MMP9 and neurological dysfunction.

MMP9 can regulate microglia-mediated inflammatory responses. however, its role in regulating microglia polarity remains to be explored. The present study aims to investigate the mechanism by which MMP9 regulates microglial polarization-mediated neuroinflammatory responses in PND caused by surgical trauma. Additionally, it seeks to delve into the underlying mechanisms of PND, with the objective of providing guidance and a foundation for the prevention and treatment of postoperative cognitive dysfunction in the aged.

## 2 Materials and methods

### 2.1 Animals

**Figure 1:**
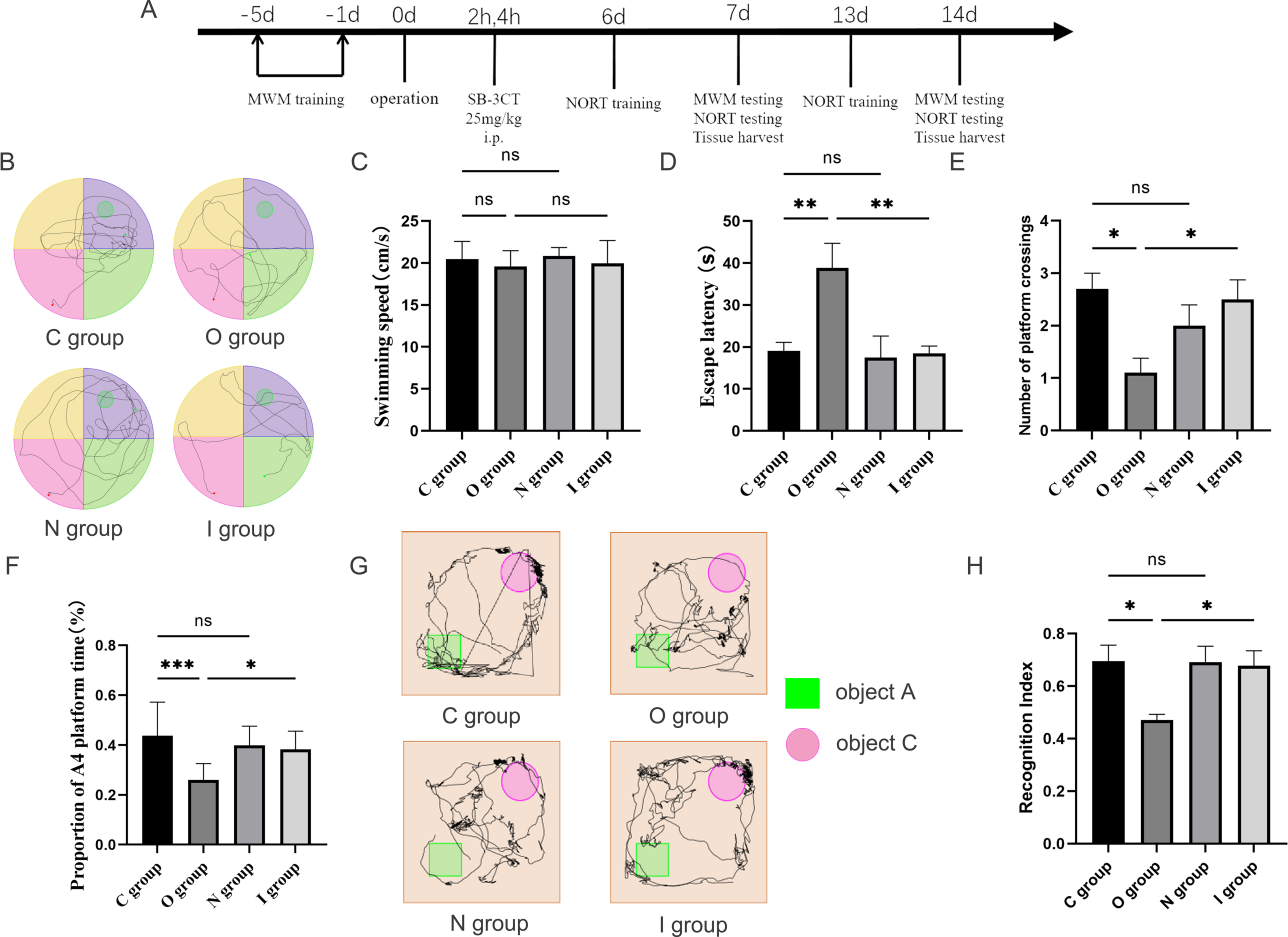
Behavioral performance at 7 days postoperatively. **(A)** Flowchart of the experiment. MWM, Morris water maze; NORT, novel object recognition task; SB-3CT, MMP9 inhibitor. Aged SD rats were subjected to MWM training for five consecutive days preoperatively. On the day of surgery, SB-3CT at a dose of 25mg/kg was injected intraperitoneally at 2h and 4h postoperatively according to the group. NORT training was performed at 6 and 13 days postoperatively. On the 7th and 14th days after the operation, 10 rats were selected for the MWM testing and the NORT testing respectively. After the behavioral tests, the hippocampus of the rats were taken for subsequent experiments. **(B)** Water maze path performance at 7 days postoperatively. **(C)** Water maze swimming speed at 7 days postoperatively (n=10). **(D)** Water maze escape latency at 7 days postoperatively (n=10). **(E)** Number of times crossing target platform area at 7 days postoperatively (n=10). **(F)** Percentage of time in the platform quadrant at 7 days postoperatively (n=10). **(G)** Novel object recognition experiment trajectory plot. **(H)** Novel object recognition experiment recognition index at 7 days postoperatively (n=10). Data are presented as the mean ± SD. *p< 0.05, **p< 0.01, and ***p< 0.001 (one-way ANOVA). ns, not significant; C group, sham operation group; O group, operation group; N group, sham operation+inhibitor group; I group, operation+inhibitor group.

The experiment was reviewed and approved by the Laboratory Animal Application Management Committee of the Center. Male Sprague Dawley (SD) rats, 8 months old, 400~500 g, were procured from Beijing Viton Lihua Laboratory Animal Technology Co., Ltd (Laboratory Animal License No. SCXK (Beijing) 2021-0006). The rats were housed in individual cages that conformed to the national standard of laboratory animal management, with temperature and humidity control, 12 h day/night alternation, and free access to food and water. After the rats were raised to 20 months old, they were randomly divided into four groups: sham operation (C) group, operation (O) group, sham operation+inhibitor (N) group, and operation+inhibitor (I) group (**Figure 1A**). In group C, only skin incision and periosteal dissection were performed. Group O underwent internal fixation of unilateral tibial plateau fracture. Skin incision and periosteal dissection were performed in group N, followed by intraperitoneal injection of 25mg/kg MMP9 inhibitor (SB-3CT) (AmBeed, A332833) as a suspension in a vehicle solution 10%DMSO+90% (20% SBE-β-CD in Saline) at 2h and 4h after the operation, respectively (22, 23). Group I underwent internal fixation of unilateral tibial plateau fracture with intraperitoneal injection of SB-3CT 25 mg/kg at 2h and 4h postoperatively. All animal experimental protocols were approved by the IACUC at the Nankai Animal Resource Center, Nankai University, and all methods were performed in accordance with the relevant guidelines and regulations.

### 2.2 Establishment of PND model

All the rats were fasted for 12 h before surgery and were anaesthetized via intraperitoneal injection of 0.9% pentobarbital sodium 50 mg/kg (China National Pharmaceutical Group, Shanghai, China). After the righting reflex disappeared, routine skin preparation of the left hind paw, disinfection and towel placement were performed. The skin was incised, and the muscle and periosteum were bluntly separated layer by layer to expose the tibia. A transverse fracture of the tibia was induced using surgical forceps, and a 0.3 mm Kirschner wire was inserted into the medullary cavity, followed by suturing. Strict aseptic operation was maintained during the operation. The incision was closed with 4–0 suture, and penicillin sodium 50 U/g (Hapharm Group Co., Ltd., Heilongjiang China) was intramuscularly injected once/d for 3 days to prevent infection. Intraperitoneal injection of Butorphanol 0.3 mg/kg (Jiangsu Hengrui Pharmaceuticals Co., Ltd., Jiangsu China) was used for postoperative analgesia. After the rats recovered from anesthesia on the thermostatic blanket, they were released to the original feeding environment.

### 2.3 Morris water maze test

The adaptive training of Morris water maze consisted of a 4-d positioning cruise experiments (spatial acquisition training) and a 1-d spatial exploration experiment. The test was conducted in a cylindrical tank with a diameter of 160 cm and a height of 60 cm, which was divided into quadrants I, II, III, and IV by four equidistant points on the wall, and a circular hidden platform was placed in the center of quadrant IV. The pool was surrounded by a reference point that remained unchanged. There was a reference around the pool that remained unchanged. Ink is poured into the water, and the platform are black to make the platform invisible. The water temperature was maintained at 21-23°C and the light in the room was constant. Each time, rats were placed into the pool from different quadrants (except the quadrant where the platform was placed) facing the wall, and if the platform was not found within 60 s, the rats were guided to the platform and stayed there for 15 s, training 4 times a day. The water maze was cleaned at the end of each training day to eliminate olfactory cues. The positioning cruise experiments were performed one day before surgery, as well as 7 and 14 days after surgery. The escape latency was the time that the rats took to board the platform. Then, the spatial exploration experiment was conducted. The platform was removed, and the rats were placed into the pool facing the wall at the midpoint of quadrant I. The escape latency and the number of times the rats crossed the original platform were recorded during the 60 s period.

### 2.4 Novel object recognition test

The experiment was divided into adaptation period, training period and testing period. 1) Adaptation period: One day before the experiment, the rats were placed in a 60cm×60cm×40cm test box and explored freely for 5 min. The rats were returned to the cage after the exploration was completed. Before each animal replacement, the feces and urine of the mice in the test box should be cleaned up and sprayed and wiped with 75% alcohol to eliminate the odor. 2) Training period: Two identical cylinders A and B were placed in the lower left and right corners of the test box. The rats were put into the test box with their backs to the two objects, and were allowed to explore the test box for 5 min freely. 3) Test period: On the second day of the modeling, the object A in the lower left corner was replaced by a new cube C. The camera installed above the test area was turned on and the rats were placed into the test box for 5 min. When the nose tip of the animal was facing or touching about 2 cm of the object range, the time of exploring object B (TB) and the time of exploring object C (TC) of the rats was recorded, and the novel object recognition index was calculated, as TC/(TB+TC)×100%.

### 2.5 Western blot

Rat brain was taken, and RIPA lysis buffer containing protease inhibitors was added to the hippocampal tissue. The supernatant was collected by centrifugation at 12,000 rpm for 20 min at 4°C. Proteins were extracted with a protein extraction kit according to the manufacturer’s protocol. After quantification of the protein concentration by a BCA Assay Kit (Beyotime, P0012), each protein sample was separated by SDS–PAGE and transferred to 0.45μm PVDF membranes. The PVDF membranes were blocked with 5% skim milk in TBST for 1 h at room temperature and then incubated overnight at 4°C with the primary antibodies. Primary antibodies included MMP9 (1:1000, Immunoway, YT1892), IL-1β (1:1000, Immunoway, YM4682) and IL-10 (1:1000, Immunoway, YT5138), IBA1 (1:1000, Immunoway, YM4765), CD86 (1:1000, SAB, 32223), ARG-1(1:1000, Immunoway, YT0311). The membranes were washed with TBST after rewarming 30 min and incubated with horseradish peroxidase-labelled secondary antibody (1:5000, SAB, L3032) for 1 h at room temperature. After thorough washing of the membranes again, the chemiluminescent signals were detected using a Super Excellent Chemiluminescent Substrate Detection Kit and Image J software was used to analyze the expression level of the target proteins by calculating the ratio of the grayscale value of the target protein band to that of the internal reference band.

### 2.6 Immunofluorescence

Rat brain tissue fixed with paraformaldehyde was subjected to dehydration in a 4°C gradient for 24 h in a solution containing 10%, 20%, and 30% sucrose. The tissue was subsequently embedded in OCT and sectioned at a thickness of 20 μm using a Leica cryostat (Leica CM1800; Leica). The pieces were washed three times at 4°C with 0.01M PBS (PH7.3) and then permeabilized with Triton X-100 (Merck, T8787) for 40 min. 5% goat serum was closed for 1 h. IBA1 (1:100, Abcam, ab283319), iNOS (1:500, Abcam, ab178945) and CD206 (1:400, Cell Signaling Technology, 24595T) primary antibodies were added of 70 μl per well, 6–8 brain slices, and incubated overnight at 4°C. After being rewarmed for 1 h, the sections were washed with PBST three times at room temperature for 5 min/time. Fluorescent secondary antibody (1:200, ab150077, Abcam; 1:200, ab150116, Abcam) was added and incubated at room temperature for 1 h under dark conditions. The sections were washed with PBS at room temperature for 3 times, 5 min/time. The sections were smoothly placed on the slides, and after being slightly dried, the pieces were sealed with anti-fluorescence attenuation tablet (C2100, Solarbio), and the glass sheet was covered for sealing. The expression of target proteins in microglia was observed under LEICA STELLARIS laser confocal microscope (Leica, Germany).

### 2.7 Flow cytometry

Rat hippocampal tissue was minced with ophthalmic scissors, homogenized by filtering through a gauze net, and then 500 μl of buffer solution was added to prepare a single-cell suspension with a concentration of 1×10^6^ cells/L after trypsin digestion. Prior to antibody labelling, the cell suspensions were incubated with FC-Receptor (Cat. #2965803, eBioscience, USA) blocking reagent at 4°C for 10 min. The cells were resuspended in a medium containing 10% fetal bovine serum and incubated with 1 ug CD86 (Cat. #200307, colne:24F, Biolegend, USA) and 1 ug CD11b (Cat. #201805, clone:OX-42, Biolegend, USA) antibodies for 30 min in the dark. The mixture was subjected to centrifugation at 1000 r/min for 5 minutes, after which the upper layer was removed. The precipitate was then thoroughly washed with PBS, and the membrane was fixed. Subsequently, 0.5 ug of CD206 fluorescent antibody (Cat. #141708, clone: C068C2, Biolegend, USA) was added and the mixture was incubated for 30 minutes in the dark. Following this incubation, the liquid was then subjected to a subsequent centrifugation at 1000 r/min for 5 minutes. Thereafter, the upper layer was extracted, and 200 ul of PBS was introduced to prepare the solution. The proportion of M1/M2 microglia was measured by multi-parameter flow cytometry. The cells were analyzed using a BD FACSCanto II instrument (Becton Dickinson Biosciences, USA). The results were analyzed by FlowJo v10.8.1 software (Beckman Coulter Biotechnology, Suzhou).

### 2.8 Statistical analyses

GraphPad Prism 9.5 software was used for statistical analysis. The Shapiro–Wilk test was used to check the normality of the data. Normally distributed measurements were expressed as mean ± standard deviation, and one-way ANOVA was used for comparison between groups. A *P* value less than 0.05 was considered to indicate statistical significance (^*^ *p*< 0.05, ^**^ *p*< 0.01, ^***^ *p*< 0.001 and ^****^ *p*< 0.0001).

## 3 Results

### 3.1 Aged rats showed cognitive decline at 7 and 14 days postoperatively, which was reversed by MMP9 inhibitors

**Figure 2:**
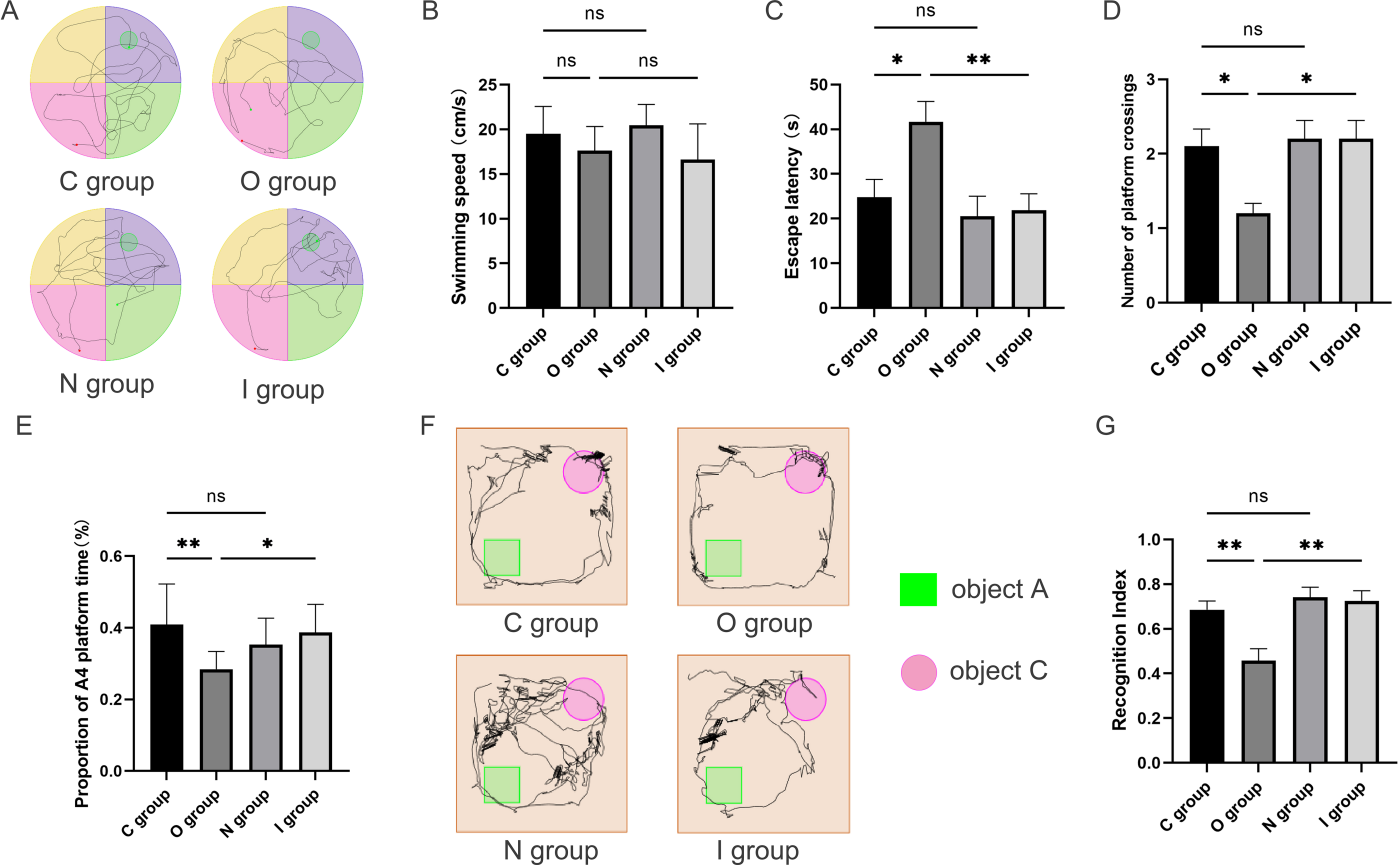
Behavioral performance 14 days after surgery. **(A)** Water maze path performance at 14 days postoperatively. **(B)** Water maze swimming speed at 14 days postoperatively (n=10). **(C)** Water maze escape latency at 14 days postoperatively (n=10). **(D)** Number of times crossing target platform area at 14 days postoperatively (n=10). **(E)** Percentage of time in the platform quadrant at 14 days postoperatively (n=10). **(F)** Trajectory map of 14-day postoperatively novel object recognition test. **(G)** Novel object recognition test recognition index on postoperative day 14 (n=10). Data are presented as the mean ± SD. *p< 0.05 and **p< 0.01 (one-way ANOVA). ns, not significant; C group, sham operation group; O group, operation group; N group, sham operation+inhibitor group; I group, operation+inhibitor group.

In this study, the Morris water maze and the novel object recognition experiments were utilized to assess the alterations in spatial orientation and learning-memory abilities of aged rats after surgery (**Figures 1B**, **2A**). The water maze test revealed no statistically significant differences among the four groups in terms of swimming speed (**Figures 1C**, **2B**), suggesting that there was no impact on the locomotor capacity of the rats among the groups. Compared with group C, there was no statistically significant difference in escape latency, target quadrant residence time, and the times of crossing target platform in groups N and I on postoperative 7 days (**Figures 1D-F**), while group O exhibited longer escape latency, fewer target quadrant residence time, and decreased times of crossing target platform conversely (**Figures 1D-F**). Compared to group O, the escape latency time in group I was reduced, the residence time in the target quadrant was increased, and the number of platform crossings was increased 7 days after surgery (**Figures 1D-F**). The findings at 14 days postoperatively of Morris water maze test were similar to those at 7 days (**Figures 2C-E**).

The novel object recognition experiment has become a popular method for measuring non-spatial memory in rats (**Figures 1G**, **2F**). Comparison with group C, the novel object recognition index of group O decreased at 7 days postoperatively (**Figure 1H**). In contrast, the novel object recognition index of group I was augmented compared with group O (**Figure 1H**). The results of the novel recognition experiment at 14 days postoperatively were consistent with those at 7 days postoperatively (**Figure 2G**). After tibial plateau surgery, the cognitive function of the aged rats was impaired, which was characterized by reduced spatial orientation and learning-memory ability, and the application of MMP9 inhibitors could reverse this effect. Overall, these findings indicated that inhibition of MMP9 could improve cognitive function postoperatively in aged rats and alleviate the neurological injury effects.

### 3.2 The expression of hippocampal inflammatory factors increased in aged rats at 7 days postoperatively and MMP9 inhibitor could reduce the neuroinflammation

**Figure 3:**
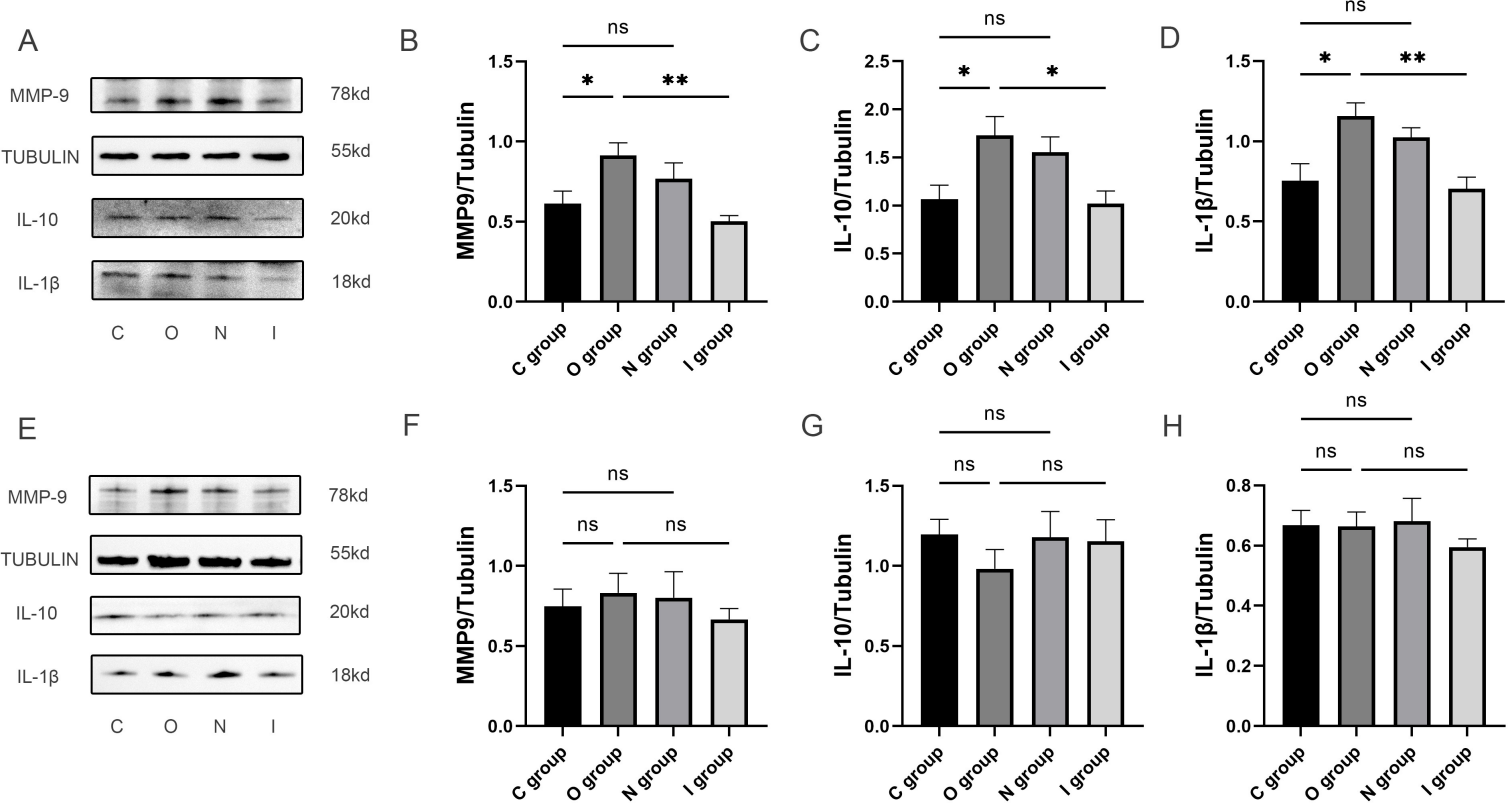
Inflammatory factor protein imprints in the hippocampus at 7 and 14 days postoperatively. **(A-D)** The quantification of inflammatory factor protein blotting images and expression levels of MMP9, anti-inflammatory factor (IL-10) and pro-inflammatory factor (IL-1β) in the hippocampus at 7 days postoperatively (n=6). **(E-H)** The quantification of inflammatory factor protein blotting images and expression levels of MMP9, anti-inflammatory factor (IL-10) and pro-inflammatory factor (IL-1β) in the hippocampus at 14 days postoperatively (n=6). Data are presented as the mean ± SD. *p< 0.05 and **p< 0.01 (one-way ANOVA). ns, not significant; C group, sham operation group; O group, operation group; N group, sham operation+inhibitor group; I group, operation+inhibitor group.

In order to investigate the impact of MMP9 on the level of neuroinflammation, Western blot was used to detect the expression levels of MMP9, IL-1β, and IL-10 in the hippocampus (**Figure 3A** and **Supplementary Material 1**). The findings revealed that the expression level of MMP9 was significantly increased in group O compared with group C at 7 days after surgery (**Figure 3B**). Furthermore, compared with group O, the expression level of MMP9 was notably reduced in group I (**Figure 3B**). Besides, the expression levels of IL-10 and IL-1β were both significantly higher in group O compared with group C at 7 days postoperatively (**Figures 3C, D**). In contrast, the expression levels of IL-10 and IL-1β in group I were significantly lower than those in group O at 7 days postoperatively (**Figures 3C, D**). While no significant difference was observed in these inflammatory factor levels at 14 days postoperatively (**Figures 3E-H**). The reduction of MMP9 expression by MMP9 inhibitor was accompanied by a concomitant reduction in IL-1β and IL-10 expression, suggesting that MMP9 can simultaneously regulate both pro-inflammatory and anti-inflammatory responses to maintain homeostasis. Therefore, combined with behavioral results, inhibition of MMP9 improved learning and memory after surgery in elderly rats by reducing the inflammatory response of CNS.

### 3.3 MMP9 inhibitor reduced microglial polarization, especially M1-type microglia

**Figure 4:**
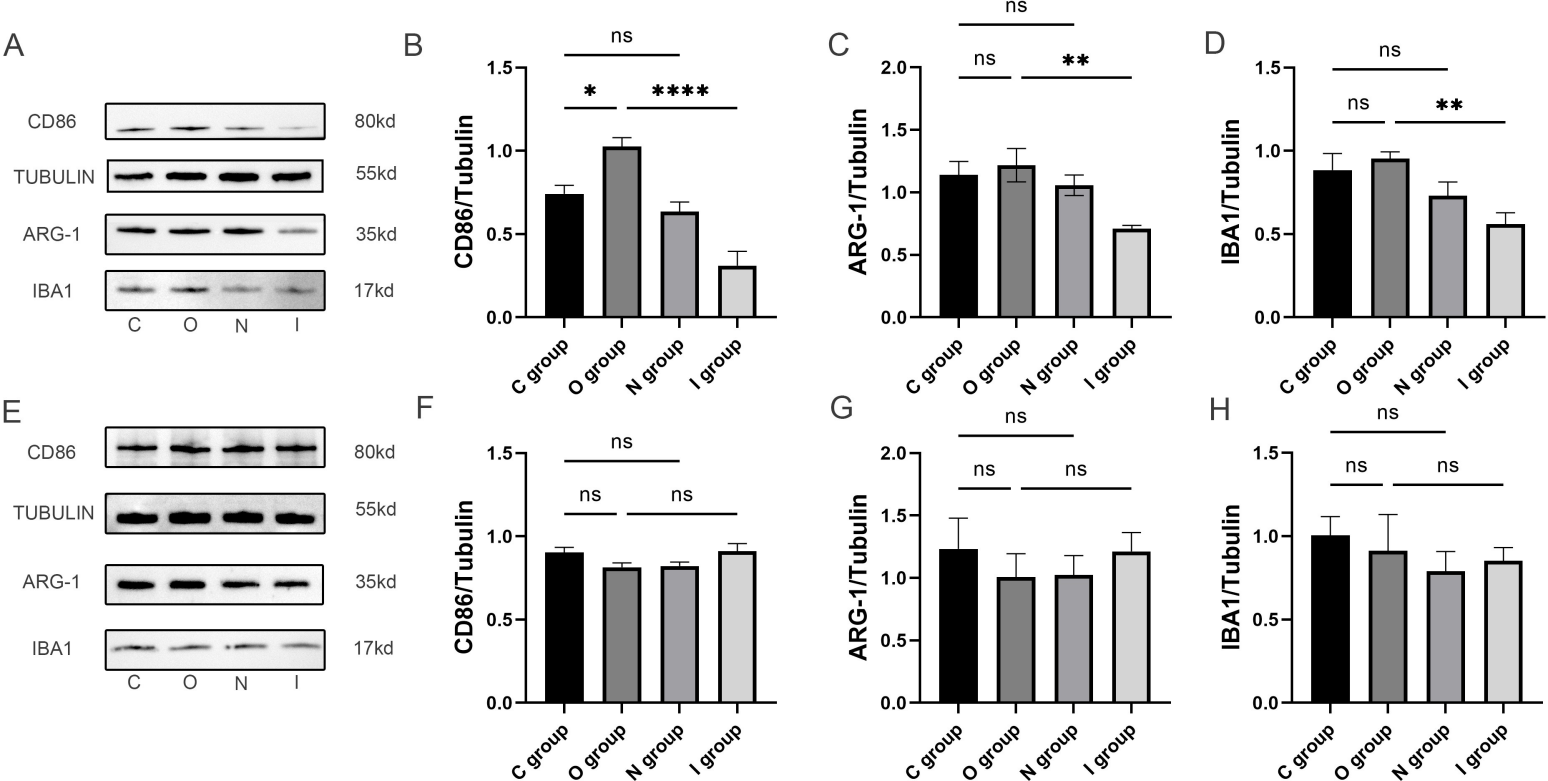
Microglia polarized protein blots in the hippocampus at 7 and 14 days postoperatively. **(A)** Representative immunoblots of microglia polarization at 7 days postoperatively. **(B-D)** Quantification of the expression levels of M1-type microglia marker (CD86), M2-type microglia marker (ARG-1) and activated microglia marker (Iba1) in the hippocampus at 7 days postoperatively (n=6). **(E)** Representative immunoblots of microglia polarization at 14 days postoperatively. **(F-H)** Quantification of expression levels of M1 microglia marker (CD86), M2 microglia marker (ARG-1) and activated microglia marker (Iba1) in hippocampus 14 days after surgery (n=6). Data are presented as the mean ± SD. *p< 0.05, **p< 0.01, and ****p< 0.0001 (one-way ANOVA). ns, not significant; C group, sham operation group; O group, operation group; N group, sham operation+inhibitor group; I group, operation+inhibitor group.

In order to investigate the mechanism of MMP9 in attenuating neuroinflammation, the expression levels of IBA1, CD86, and ARG-1 in the hippocampal tissues were detected by Western blot (**Figure 4A**). There was no statistically significant difference in IBA1 between group C and group O (**Figure 4D**). The expression level of IBA1 was significantly lower in group I at 7 days postoperatively in comparison with group O (**Figure 4D**), thereby suggesting that the inhibition of MMP9 attenuated microglial activation. The results demonstrated that compared with group C, the expression level of CD86 in group O increased significantly 7 days after surgery (**Figure 4B**), indicating that the surgery mainly induced the activation of M1-type microglia. Meanwhile, the expression levels of CD86 and ARG-1 in group I were significantly lower than those compared with group O (**Figures 4B, C**), among which the effect of MMP9 inhibitor on the expression of CD86 was more prominent, further indicating that MMP9 inhibitor could reduce the activation of microglia, primarily the M1 phenotype. No statistically significant differences were observed in the expression levels of IBA1, CD86, and ARG-1 in the hippocampal tissues of each group 14 days after surgery (**Figures 4E-H**). The above evidence elucidated that the proliferation of microglia in the hippocampus caused by acute surgical trauma could be reversed by the application of MMP9 inhibitor, especially M1-type microglia.

**Figure 5:**
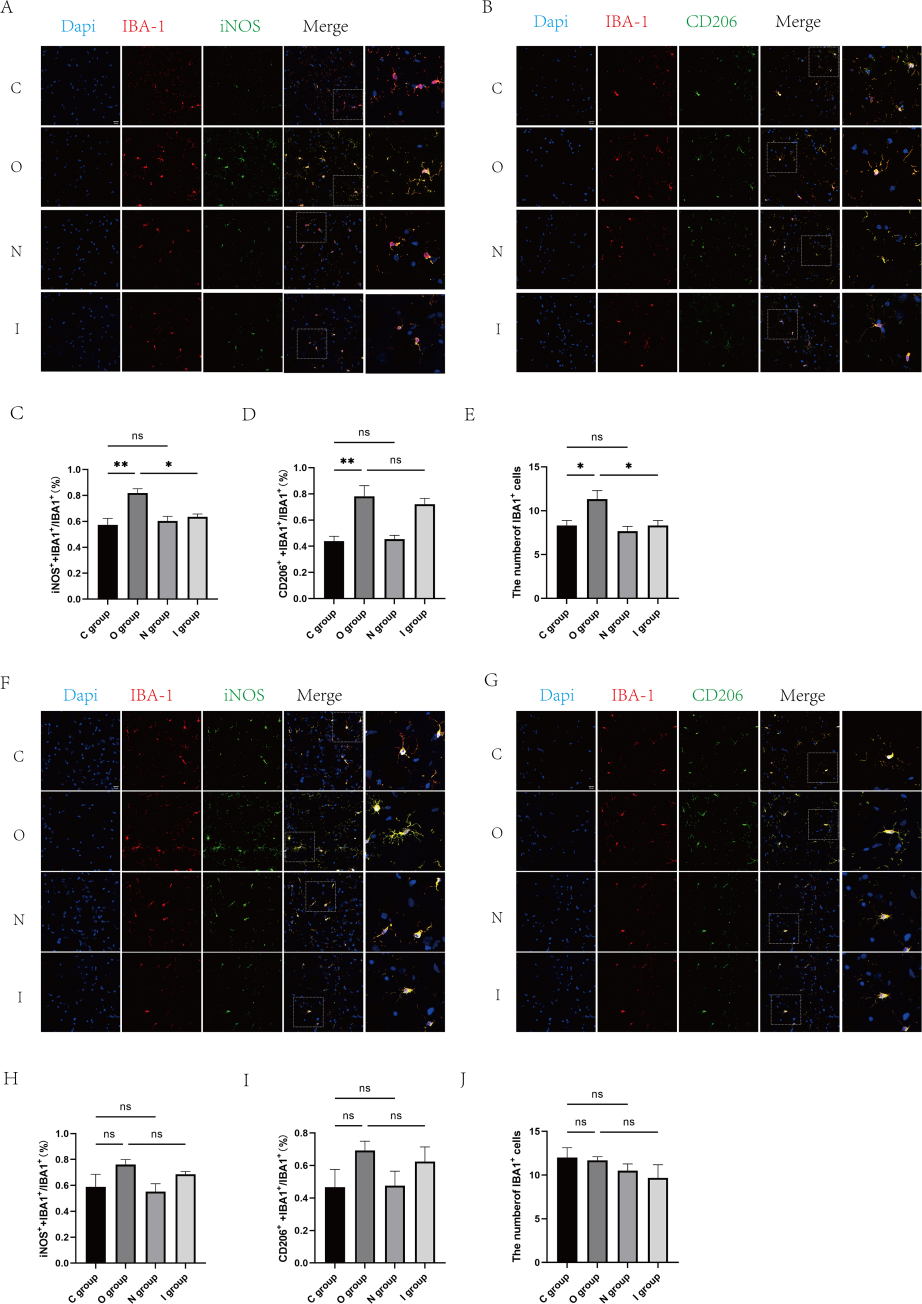
Fluorescent staining of the hippocampal CA1 region at 7 and 14 days postoperatively. **(A)** Double immunofluorescence staining of microglia (IBA-1, red) with M1 markers (iNOS, green) in the CA1 region of the hippocampus at 7 days postoperatively. Nuclei were stained with Dapi. Scale bar: 20 μm. **(B)** Double immunofluorescence staining of microglia (IBA-1, red) in the CA1 area of the hippocampus with M2 marker (CD206, green) at 7 days postoperatively. Cell nuclei were stained with Dapi. Scale bar: 20 μm. **(C)** Proportion of M1-type microglia to activated microglia in the CA1 region of the hippocampus 7 days after surgery (n=3). **(D)** Proportion of M2-type microglia to activated microglia in the CA1 region of the hippocampus at 7 days postoperatively (n=3). **(E)** Number of activated microglia in the hippocampal CA1 region at 7 days postoperatively (n=6). **(F)** Double immunofluorescence staining of microglia (IBA-1, red) with M1 markers (iNOS, green) in the hippocampal CA1 region at 14 days postoperatively. Cell nuclei were stained with Dapi. Scale bar: 20 μm. **(G)** Double immunofluorescence staining of microglia (IBA-1, red) in the CA1 area of the hippocampus with M2 marker (CD206, green) at 14 days postoperatively. Cell nuclei were stained with Dapi. Scale bar: 20 μm. **(H)** Proportion of M1-type microglia to activated microglia in the hippocampal CA1 region 14 days after surgery. **(I)** Proportion of M2-type microglia to activated microglia in the CA1 region of the hippocampus 14 days after surgery (n=3). **(J)** Number of activated microglia in the CA1 region of the hippocampus 14 days after surgery (n=6). Data are presented as the mean ± SD. *p< 0.05 and **p< 0.01 (one-way ANOVA). ns, not significant; C group, sham operation group; O group, operation group; N group, sham operation+inhibitor group; I group, operation+inhibitor group.

Meanwhile, in order to further clarify the polarization of microglia in the hippocampus, immunofluorescence staining was performed to count M1 and M2 type microglia (**Figures 5A, B**). The results showed that the proportion of M1-type microglia and M2-type microglia was significantly increased in group O than in group C 7 days after surgery (**Figures 5C-E**). Conversely, the proportion of M1-type microglia was significantly lower in group I than in group O (**Figure 5C**). At the 14-day postoperative interval, no statistically significant differences were observed in the proportion of M1 and M2 microglia across the groups (**Figures 5F-J**). As a result, the MMP9 inhibitor suppressed the activation of microglia after surgery, primarily M1-type microglia.

Based on the above findings, the results demonstrated that MMP9 was indeed involved in microglia-mediated neuroinflammatory responses, and MMP9 inhibitor could ameliorate these effects by regulating the polarization of microglia to enhance the secretion of inflammatory factors.

### 3.4 MMP9 inhibitor attenuated surgical-induced inflammation by reducing the proportion of activated microglia

**Figure 6:**
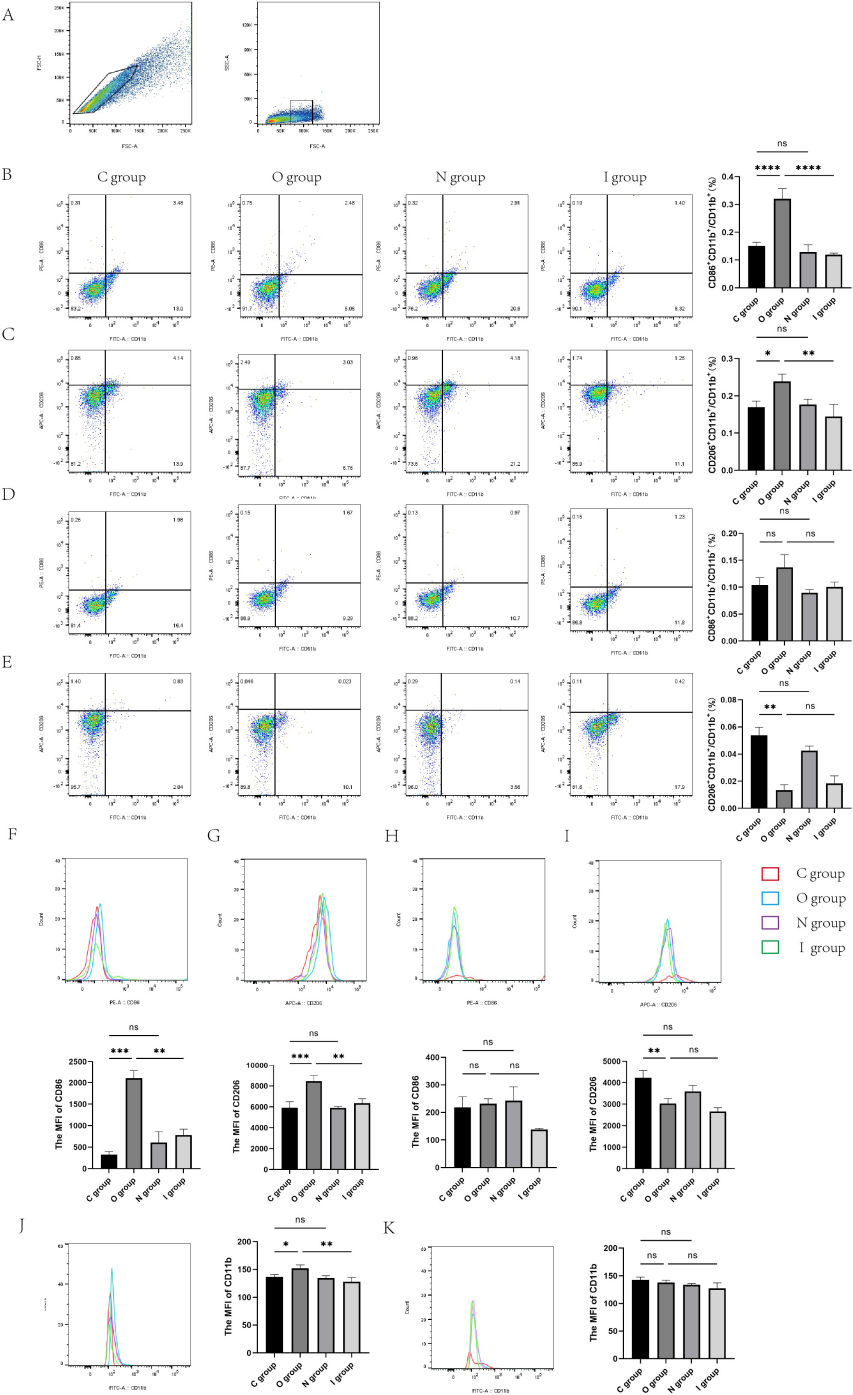
Flow cytometry of the rat hippocampus at 7 and 14 days postoperatively. **(A)** The gating strategy for microglia. Microglia cells within the gate were used for CD86/CD206 analysis. **(B)** Proportion of M1-type microglia in the hippocampus of rats at 7 days postoperatively (n=3). **(C)** Proportion of M2-type microglia 7 days after surgery (n=3). **(D)** Proportion of M1-type microglia in the hippocampus of rats at 14 days postoperatively (n=3). **(E)** Proportion of M2-type microglia 14 days after surgery (n=3). **(F)** Representative histogram of mean fluorescence intensity (MFI) of CD86–7 days after surgery (n=3). **(G)** Representative histogram of MFI of CD206–7 days after surgery (n=3). **(H)** Representative histogram of MFI of CD86 at 14 days postoperatively (n=3). **(I)** Representative histogram of MFI of CD206 at 14 days postoperatively (n=3). **(J)** Representative histogram of mean fluorescence intensity (MFI) of CD11b 7 days after surgery (n=3). **(K)** Representative histogram of MFI of CD11b at 14 days postoperatively (n=3). Data are presented as the mean ± SD. *p< 0.05, **p< 0.01, ***p< 0.001, and ****p< 0.0001 (one-way ANOVA). ns, not significant; C group, sham operation group; O group, operation group; N group, sham operation+inhibitor group; I group, operation+inhibitor group.

To verify the specific number of activated microglia cells, flow cytometry was performed using a variety of microglial markers to assess the M1 and M2 phenotypes of the rat hippocampus. CD11b^+^ initially served as a marker for microglia cells (**Figure 6A** and **Supplementary Material 2**). Then, the mean fluorescence intensities (MFI) of CD86^+^ (M1 marker) and CD206^+^ (M2 marker) were detected. The results demonstrated that the percentage of rat hippocampal M1-type microglia and M2-type microglia were significantly increased in group O than in group C on the 7th day after surgery (**Figures 6B, C, E, G, J**). Compared with group O, the percentage of M1-type microglia and M2-type microglia almost returned to baseline levels in group I at 7 days after surgery (**Figures 6B, C, F, G**), with M1 microglia declining more significantly after application of inhibitors (**Figure 6B**). In contrast to group C, there was no statistically significant difference in the activation percentage of M1 microglia in group O at 14 days postoperatively (**Figures 6D, H**). However, compared with group C, the percentage of M2 microglia was significantly lower in group O at 14 days postoperatively (**Figures 6E, I**). Group I identically demonstrated similar activation percentage of both M1 and M2 microglia compared to group O on postoperative day 14 (**Figures 6D, E, H, I, K**). Taken together, these findings suggested that the surgery caused the activation of microglia, which polarized into M1/M2 microglia. After the application of MMP9 inhibitors, the percentage of activated microglia significantly decreased, with a predominance of M1 microglia. Western blotting analysis and immunofluorescence staining also verified the significant decrease of M1-type microglia in the hippocampus of group I compared with group O. Collectively, these results indicated that MMP9 inhibitor could regulate the excessive activation of microglia caused by surgery, especially M1-type microglia, thereby exerting neuroprotective effects.

## 4 Discussion

Neuroinflammation, leading to abnormal neuronal structure and function or even death, is an important factor causing postoperative neurodegenerative pathologies. The present study focused on the effects of MMP9-mediated microglial polarization on cognitive function in perioperative aged rats. This results showed that surgical stimulation induced an increase in the level of MMP9, which stimulated the polarization of microglia and aggravated hippocampal neuroinflammation. In contrast, the inhibition of the MMP9 activity reduced the polarization of microglia, attenuated neuroinflammation, and improved the cognitive function of the rats.

The development of preventive and treatment methods for PND has been hindered by numerous challenges, many of which stem from the incompleteness regarding its pathogenesis. As a major inflammatory mediator, MMP9 plays a crucial role in the regulation of the neuroinflammation (19). There is growing evidence that anesthesia/surgical stimulation increases TNF-α and IL-1β levels in the hippocampus, and the levels decrease after regulating MMPs through toll-like receptors (24, 25). Similarly, the current study revealed that surgical stimulation resulted in increased IL-1β in the hippocampus, and IL-1β fell back to baseline levels in the control group after the use of MMP9 inhibitor at 7 days postoperatively. This finding is consistent with the known ability of MMP9 to activate pro-IL-1β (26) and the role of IL-1β in driving M1 polarization (27), suggesting that this may be an important mechanism by which MMP9 promotes microglia polarization.

Available evidence suggests that neuroinflammation is closely associated with cognitive impairment caused by anesthesia/surgery. Microglia, a major source of pro-inflammatory factors, plays a crucial role in neuroinflammation (12, 13). Microglial activation is amplified and prolonged in the aged brain compared to adults (28). Given the high incidence of cognitive impairment following orthopedic surgery in the geriatric (29), we selected open reduction and internal fixation for tibial fracture as our model. Notably, in contrast to group C, the percentage of M2 phenotype in the hippocampus decreased in group O 14 days after the surgery. These data in our study support that aged microglia exhibit heightened sensitivity to damage signals, resulting in early-onset and sustained M1 polarization, whereas M2 polarization is transient and functionally impaired (30).

Microglia can induce activation of astrocytes, jointly causing neuronal toxicity and death (12). The pathophysiological changes associated with anesthesia/surgery have been shown to induce microglial polarization and proliferation, particularly of M1-type microglia (31). Consistent with our findings, compared with group C, the expression level of CD86 and iNOS^+^+IBA1^+^/IBA1^+^ microglia in group O increased significantly 7 days after surgery, indicating that the surgery mainly induced the activation of M1-type microglia. Microglia are furthermore involved in cholesterol clearance and influence the inflammatory environment by regulating lipid metabolism. Elevated MMP9 activity disrupts the lipid exchange between astrocytes and microglia, leading to cholesterol accumulation and inducing microglia M1-related inflammatory responses. Recently, it was reported that MMP9 inhibitor SB-3CT treatment reduced astrocytic and microglial reactivity, which might have contributed to the regulation of cholesterol and lipid homeostasis, thereby mitigating neuroinflammation (32). This is also the potential mechanism by which MMP9 regulates microglial polarization identified in our current study.

MMP9 is an important target in the regulation of microglial polarization, which is critical for transmission of neuroinflammation. The present study demonstrated that MMP9 inhibitors inhibited the proliferation of microglia generated by surgical stimulation 7 days after surgery, especially M1-type microglia, leading to a decrease in pro-inflammatory cytokine IL-1β. This implies that MMP9 can enhance the neuroinflammation after surgery, contributing to the cognitive decline and MMP9 inhibition promotes the rebalancing of inflammatory factor production. However, these changes in inflammation were not detected at 14 days postoperatively. This result suggests that the neuroinflammation caused by surgery is an acute and self-limiting pathological process. It has been reported equally that acute inflammatory responses are self-limiting (33, 34), related to pro-resolving lipid mediators, which avoids causing persistent nerve damage. The inflammatory response is a dynamic process. Our study only observed changes in neuroinflammation at 7 and 14 days postoperatively, possibly missing the differences in intervention at the early (inflammatory peak) and late (chronic inflammation) stages. Based on the results obtained 7 days after surgery, the MMP9 inhibitor was effective in reducing neuroinflammation and alleviating cognitive dysfunction. Acute inflammatory responses and changes in microglia phenotype, even transient, have still profound effects on the neurological microenvironment, synaptic plasticity and long-term cognitive recovery (35, 36). Although there was no significant differences in inflammatory levels and microglial polarization among the groups 14 days after the surgery, the behavioral tests conducted 14 days after the surgery revealed that group I was superior to group O. Injury is a key driver of inflammation, a critical and necessary response involving multiple mediators aimed to restore tissue homeostasis (37). Ordinary surgery can overwhelm the immune system, trigger neurological complications, and even lead to Alzheimer’s disease. It has been suggested in the literature that compared with healthy elderly individuals, those who have undergone surgery/anesthesia are more likely to develop Alzheimer’s disease as a result of PND (38). Therefore, it is essential to control acute inflammation after surgery and intervention is necessary irrespective of its self-limiting characteristic.

A well-functioning blood-brain barrier is crucial to maintaining functional homeostasis of the CNS. MMP9 not only degrades tight junctions, but also is cytopathic to pericytes and upregulated transcytosis, leading to blood-brain barrier hyperpermeability (39). At the same time, inflammatory peripheral cells migrates to the CNS which release LPS and IFN-γ, thereby activating the TLR4/NF-κB pathway of microglia and inducing the polarization of M1 microglia (40). Hence, MMP9 regulates microglial polarization by modulating inflammatory factors and inflammatory signaling pathways. MMP9 also changes the microenvironment of the CNS by destroying the blood-brain barrier. Restoration of blood-brain barrier function by inhibition of MMP9 is effective in preventing neuroinflammation and improving cognitive behavior. In addition, MMP9 indirectly regulates microglial polarization by influencing astrocytes. Elevated MMP9 could disrupt homeostasis of the water channel aquaporin-4 (AQP4) of astrocytes by cleaving β-dystroglycan, a key anchor protein (41). Inhibition of MMP9 can restore AQP4 polarity and improve lymphatic drainage while promoting M2 polarization of microglia (32).

In the PND model, surgical trauma induces the metabolism of microglia to shift from oxidative phosphorylation to glycolysis, promoting M1-type polarization (42). The activation of MMP9 may further promote the polarization of microglia towards M1 type by influencing cellular metabolic pathways. This is primarily the study topic that our research team is currently engaged in. Surgical interventions elevate reactive oxygen species levels, which in turn activates MMP9, leading to the shadding of the receptor for advanced glycation end products (RAGE). The shedding of RAGE further activates NF-κB, thereby promoting the secretion of pro-inflammatory cytokines. These cytokines are able to activate microglia, causing them to polarize towards the M1 type (43), which amplify neuroinflammation by secreting additional mediators, establishing a self-sustaining feedback loop that exacerbates neuronal dysfunction. Aberrant microglial activation results in excessive production of pro-inflammatory cytokines, oxidative stress, and synaptic disruptions, which collectively contribute to PND-associated cognitive deficits.

Above all, MMP9 dynamically regulates the M1/M2 polarization of microglia through mechanisms including activation of inflammatory pathways, lipid metabolism, blood-brain barrier disruption, and AQP4 polarity regulation. We particularly linked these possibilities to our experiment observations. We explicitly found that there might be an obvious relationship between the massive accumulation of MMP9 and microglial polarization. Targeting MMP9 can reverse neuroinflammation and provide a new strategy for the treatment of neurodegenerative diseases. In the future, it is necessary to further clarify the specific regulatory network of MMP9 on different microglial subtypes.

It should be noted that there are several limitations to the present experiment. Firstly, we only studied changes of neuroinflammation and phenotypes of microglia in the hippocampus, the main brain region responsible for learning and spatial memory, and did not explore multiple cognitive related brain regions such as the midbrain, striatum, and amygdala, which may result in the lack of coherence and connectivity in our research. The evidence for the large-scale coordination of the medial prefrontal cortex with the hippocampus and posterior parietal cortex as a neural mechanism supporting spatial learning has been confirmed (44). The study, involving brain regions such as the prefrontal cortex, will provide a more comprehensive understanding of the cognitive network. Secondly, recent advancements in the field have suggested that the conventional microglial polarization categorization, namely M1 and M2 states, should be regarded as a continuous process of transition rather than two discrete extreme states (14). Microglial research has been constrained by a rolling series of dichotomies (resting versus activated and M1 versus M2). The observed changes in markers of the present study should be interpreted as a evidence of the shift in the overall functional state of microglia towards pro-inflammatory or anti-inflammatory. To elucidate the complexity and diversity of microglia in neurological diseases, novel experimental methods, such as isotope labeling technique and single-cell cytometey, are anticipated to be employed in microglial heterogeneity. Thirdly, SB-3CT can inhibit MMP2, although it is generally considered to exhibit higher potency and selectivity for MMP9 (IC_50_ **≈**20 nM) (45). We cannot completely rule out the contribution of MMP2 inhibition to the observed effects. Significantly, our current analysis mainly focuses on the expression level of the MMP9 protein. This study did not directly evaluate the enzyme activity of MMP9, and the activity assay can more directly and accurately evaluate the function of the inhibitor. Future studies using gene knockout techniques and specific MMP9 activity detection could further delineate the specific roles. Fourthly, this experiment did not demonstrate a transition node for inflammation and microglial polarization changes between 7 and 14 days. Future research should explore this turning point, which will provide a more comprehensive understanding of the PND induced by surgery to reduce the duration of postoperative cognitive decline through effective measures.

## 5 Conclusion

In summary, our study elucidates the mechanism of MMP9 on cognitive function after surgery in the elderly. This study improves our understanding of the roles of MMP9, which serve as a mediator for microglial polarization, leading to exacerbated neuroinflammation and subsequent cognitive impairment. Additionally, MMP9 inhibition has been observed to impede polarization of M1 microglia, exerting neuroprotective effects. These findings underscore the potential of MMP9 as a therapeutic target for the prevention and treatment of postoperative cognitive impairment.

## Data Availability

The raw data supporting the conclusions of this article will be made available by the authors, without undue reservation.
