# Supplementary material for: The critical role of matrix metalloproteinase 9-mediated microglial polarization in perioperative neurocognitive disorders of aged rats
Source: Front Immunol. 2025 Aug 21;16:1650254. doi: 10.3389/fimmu.2025.1650254 (PMC12408272; doi:10.3389/fimmu.2025.1650254)
Supplement: Supplementary file 1 [file Table1.docx]

**Original images of Western blot analysis.**

**Images in Figure 3 A**

**MMP9**

**
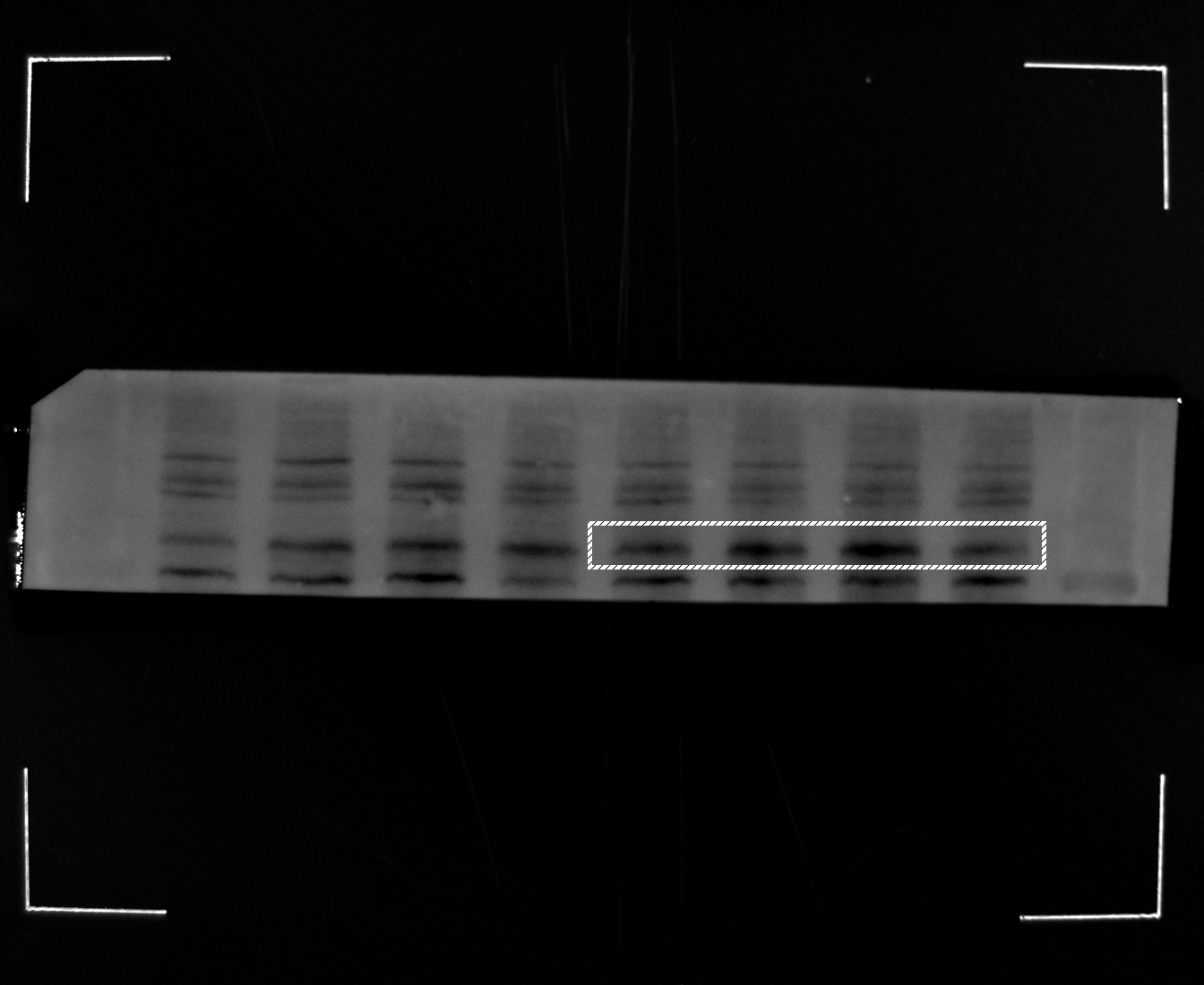
**

78kd

**C O N I**

**TUBULIN**

**
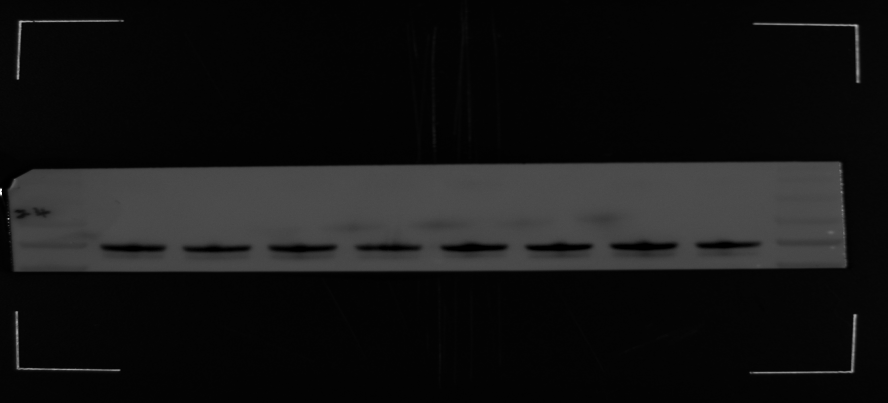
**

55kd

**C O N I**

**IL-10**


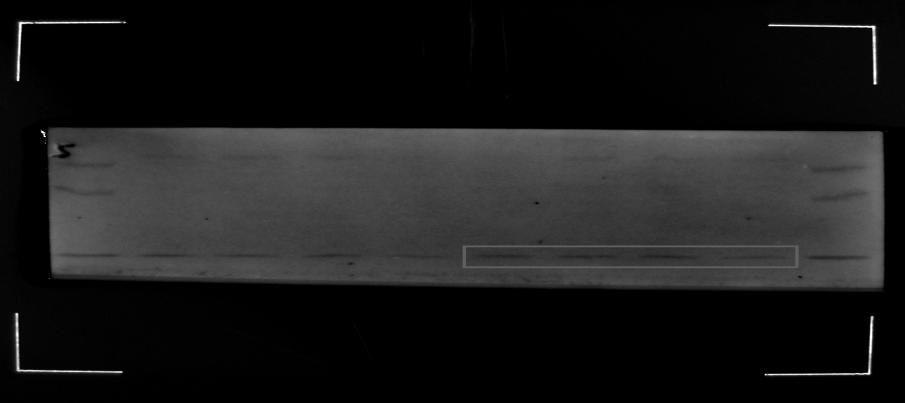


20kd

**C O N I**

**IL-1β**

**
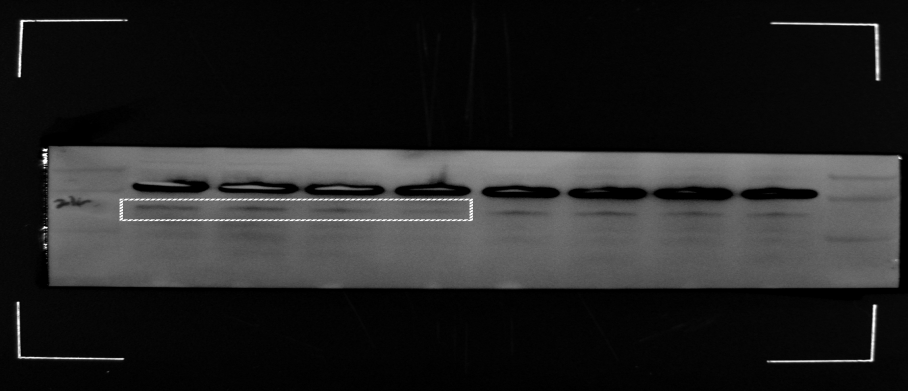
**

18kd

**C O N I**

**Images in Figure 3 E**

**MMP9**

**
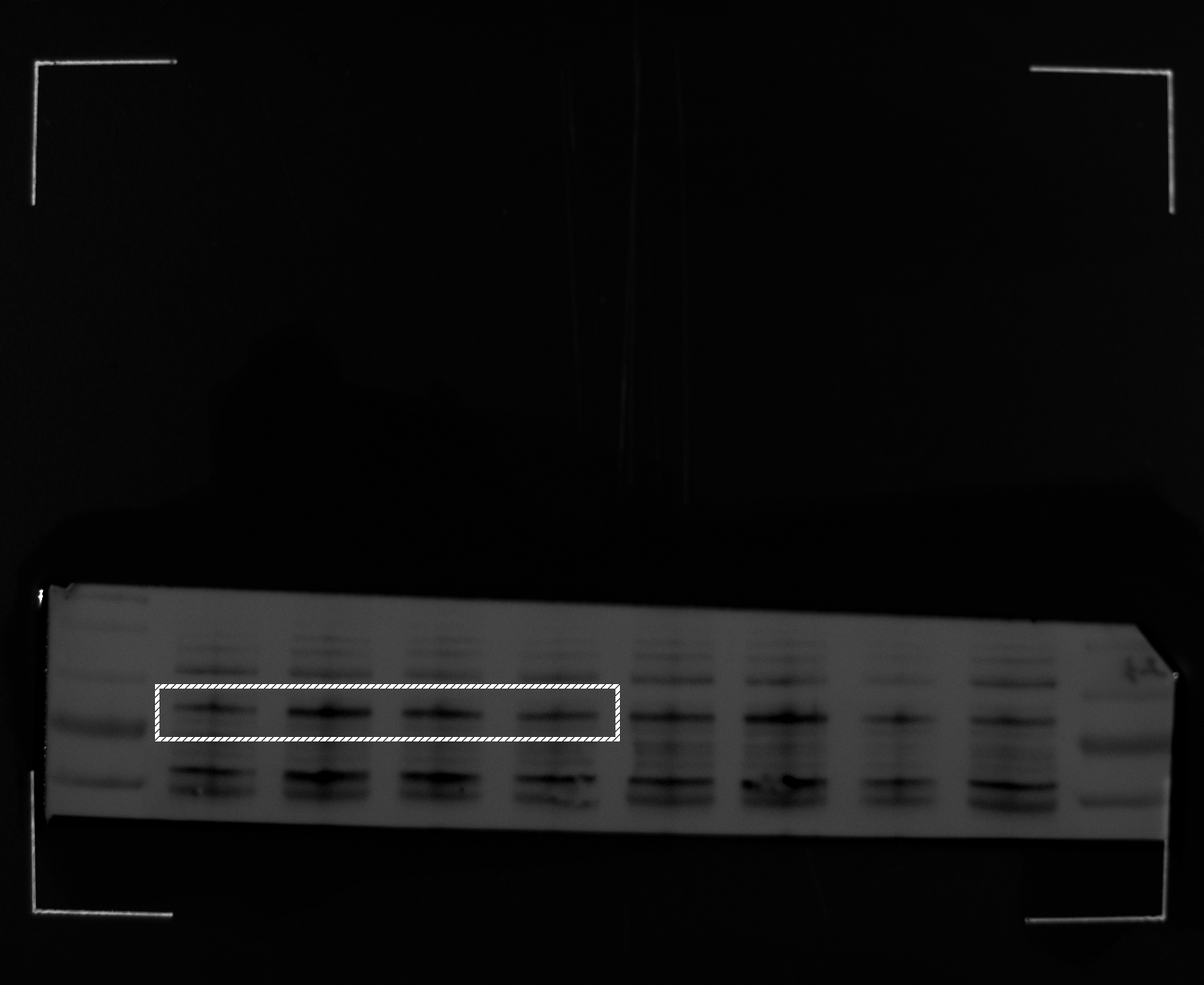
**

78kd

**C O N I**

**TUBULIN**

**
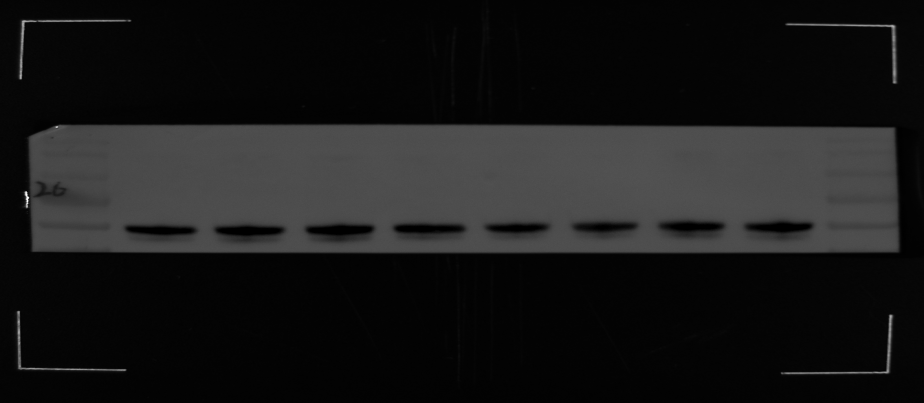
**

55kd

**C O N I**

**IL-10**

**
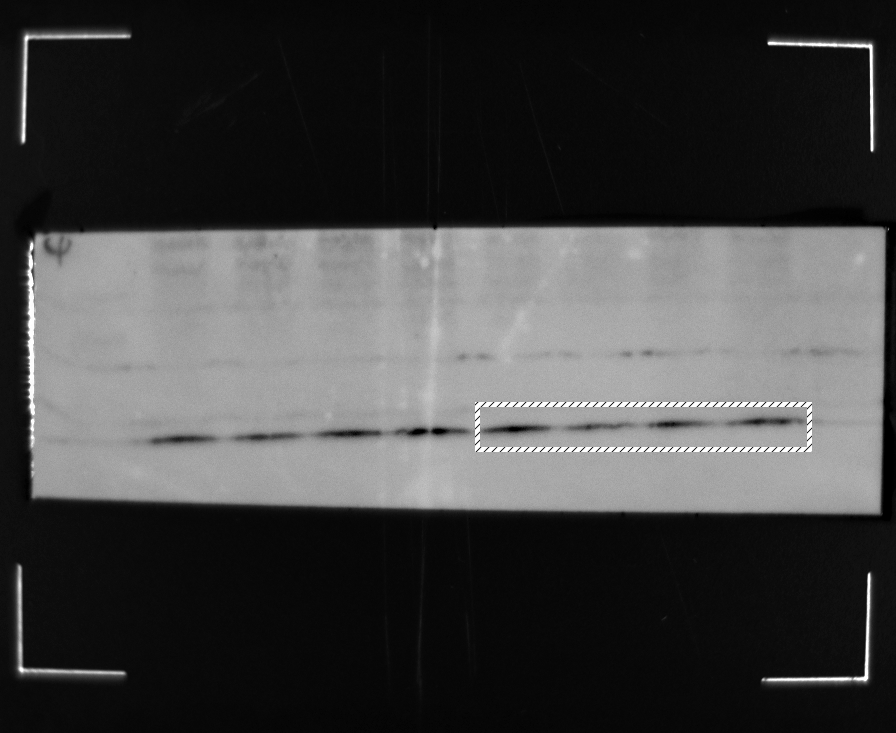
**

20kd

**C O N I**

**IL-1β**

**
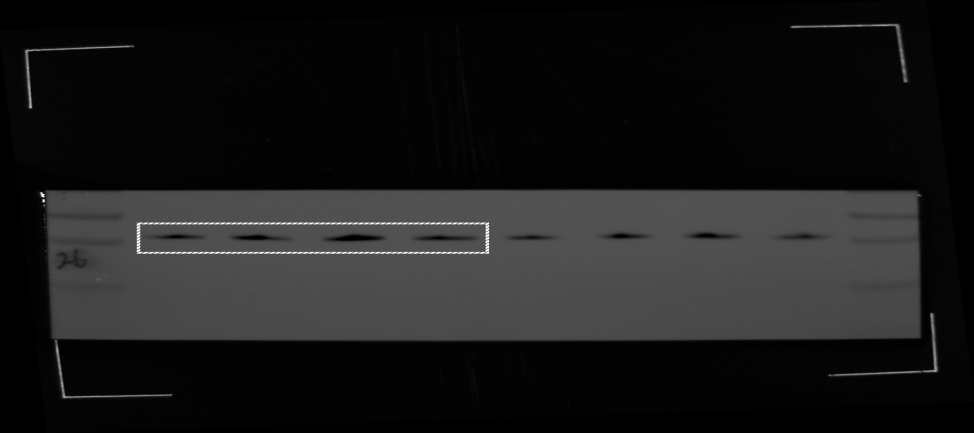
**

18kd

**C O N I**

**Images in Figure 4 A**

**CD86**


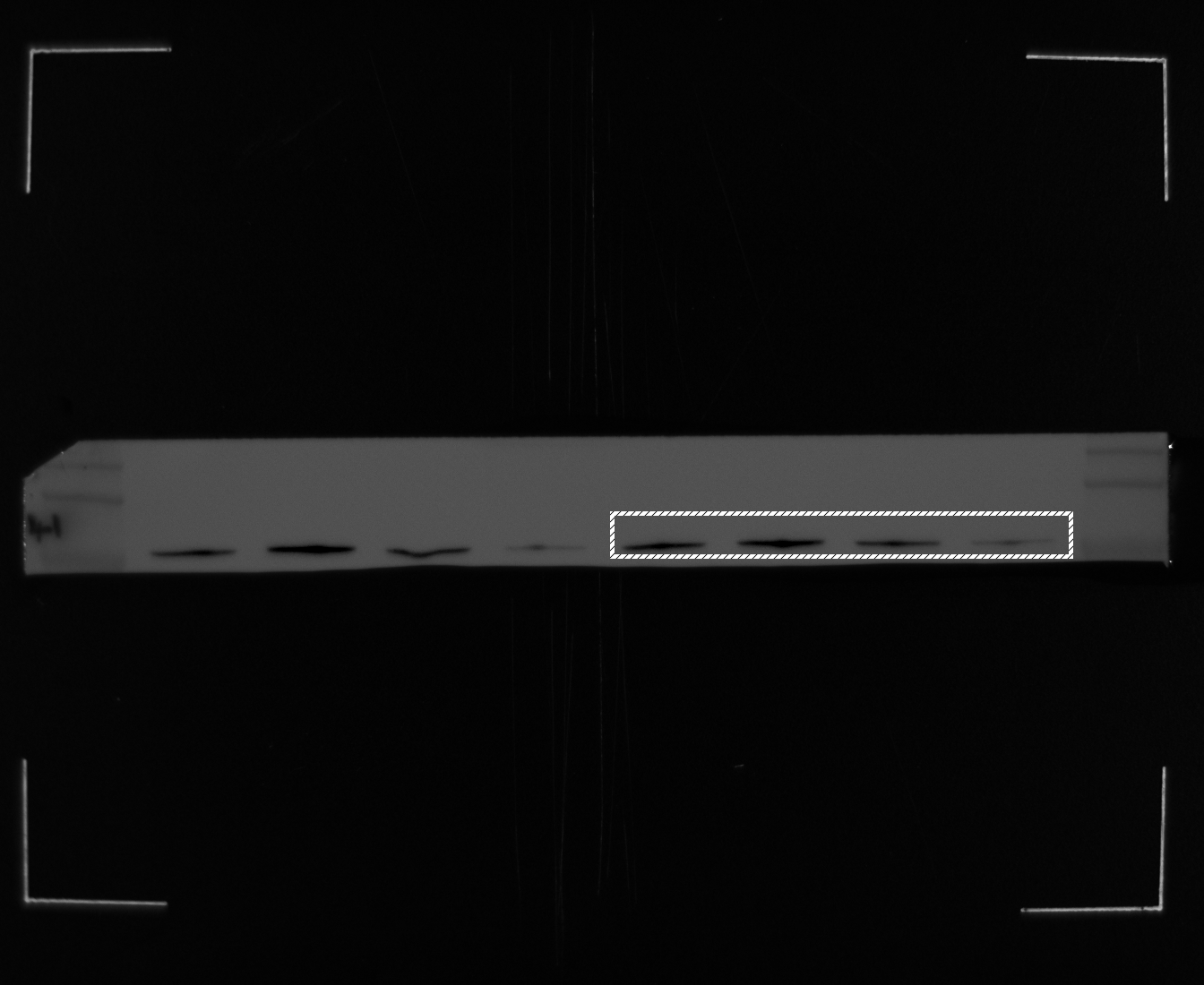


**C O N I**

80kd

**TUBULIN**

**C O N I**

**
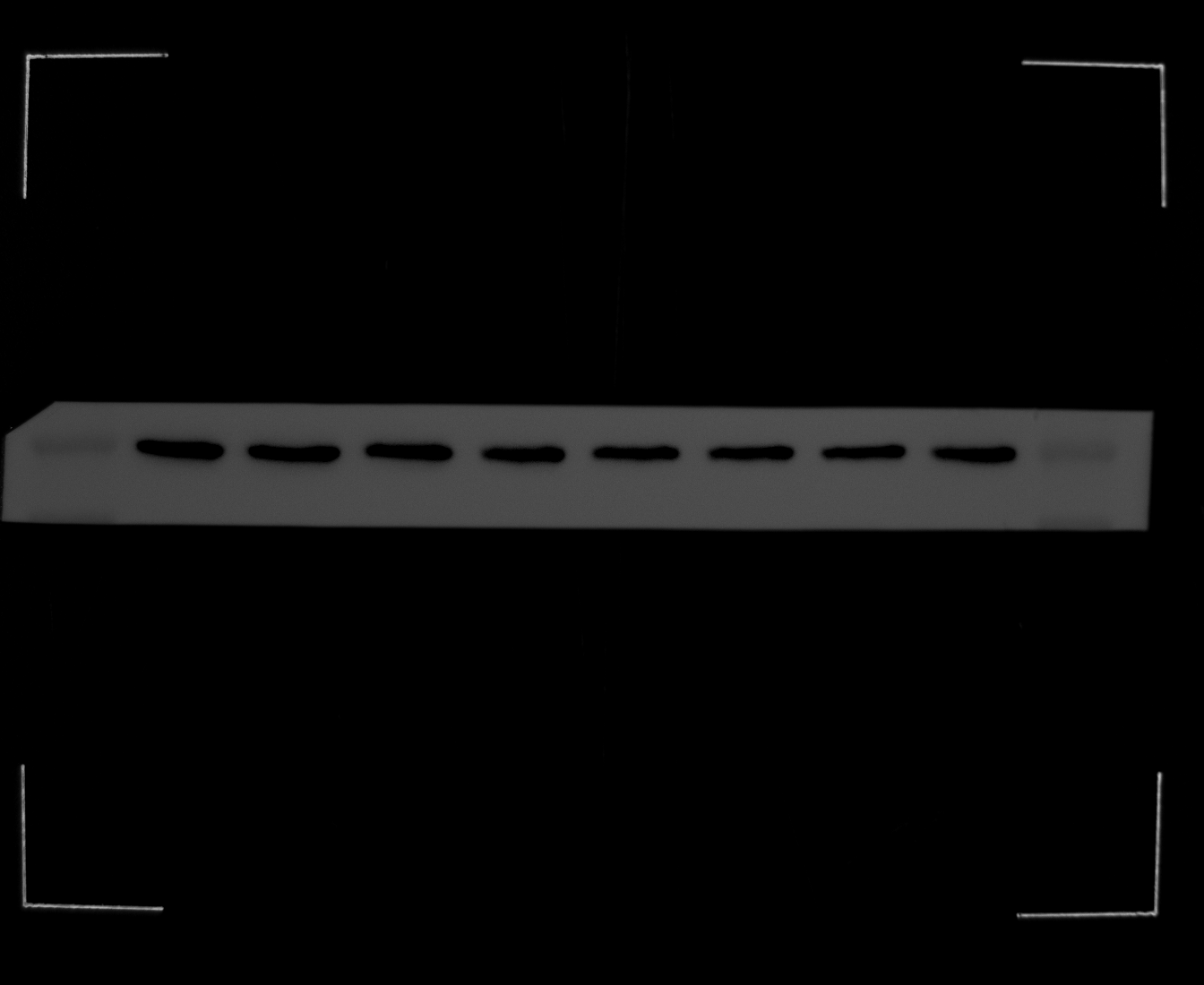
**

55kd

**ARG-1**

**
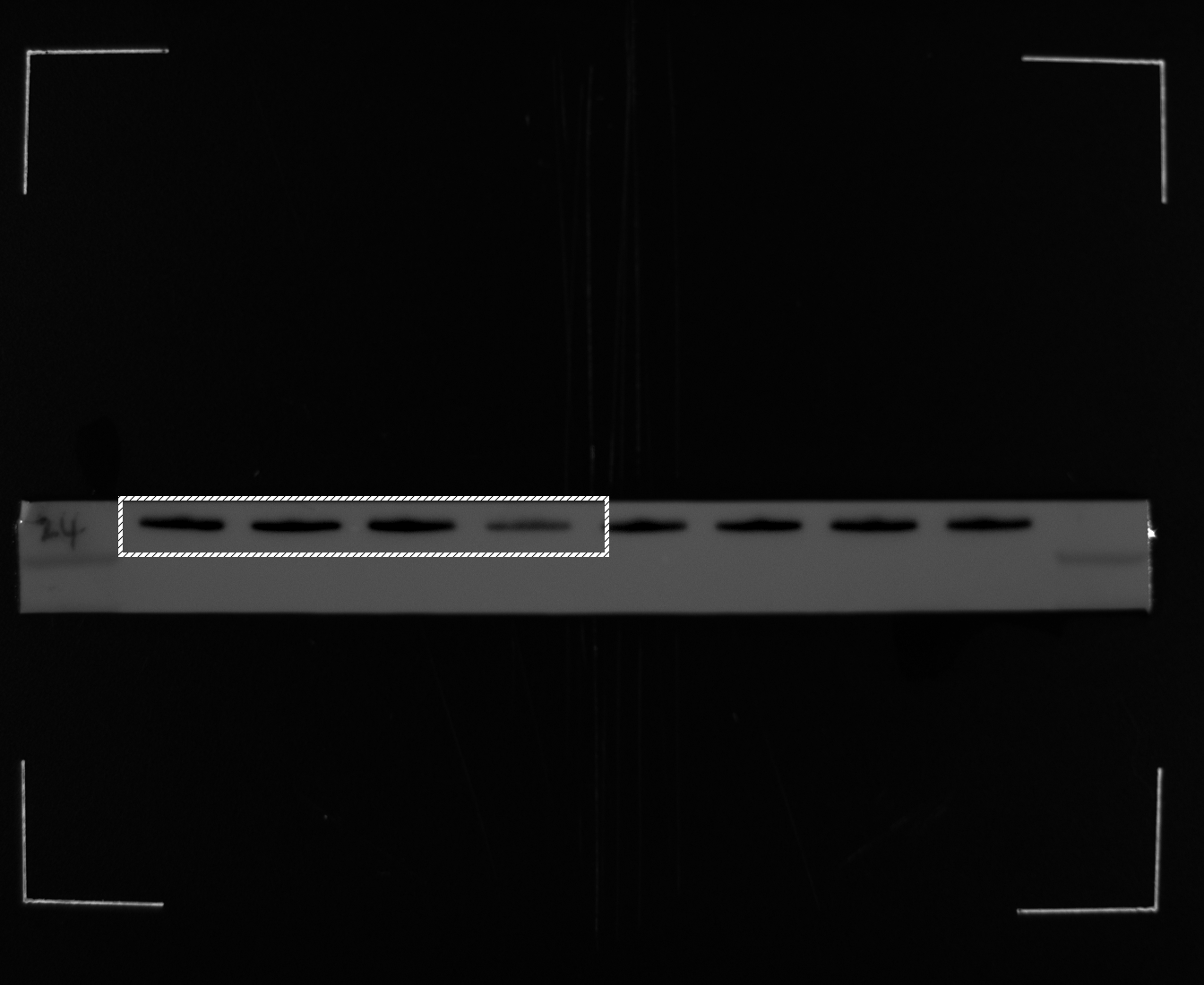
**

35kd

**C O N I**

**IBA1**

**
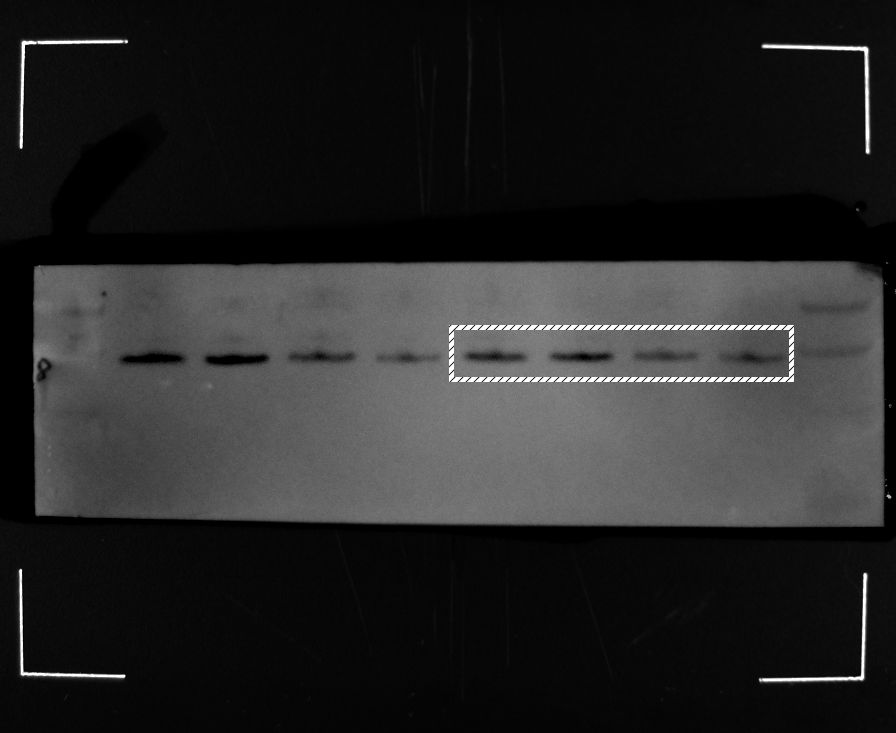
**

**C O N I**

17kd

**Images in Figure 4 E**

**CD86**

**C O N I**

**
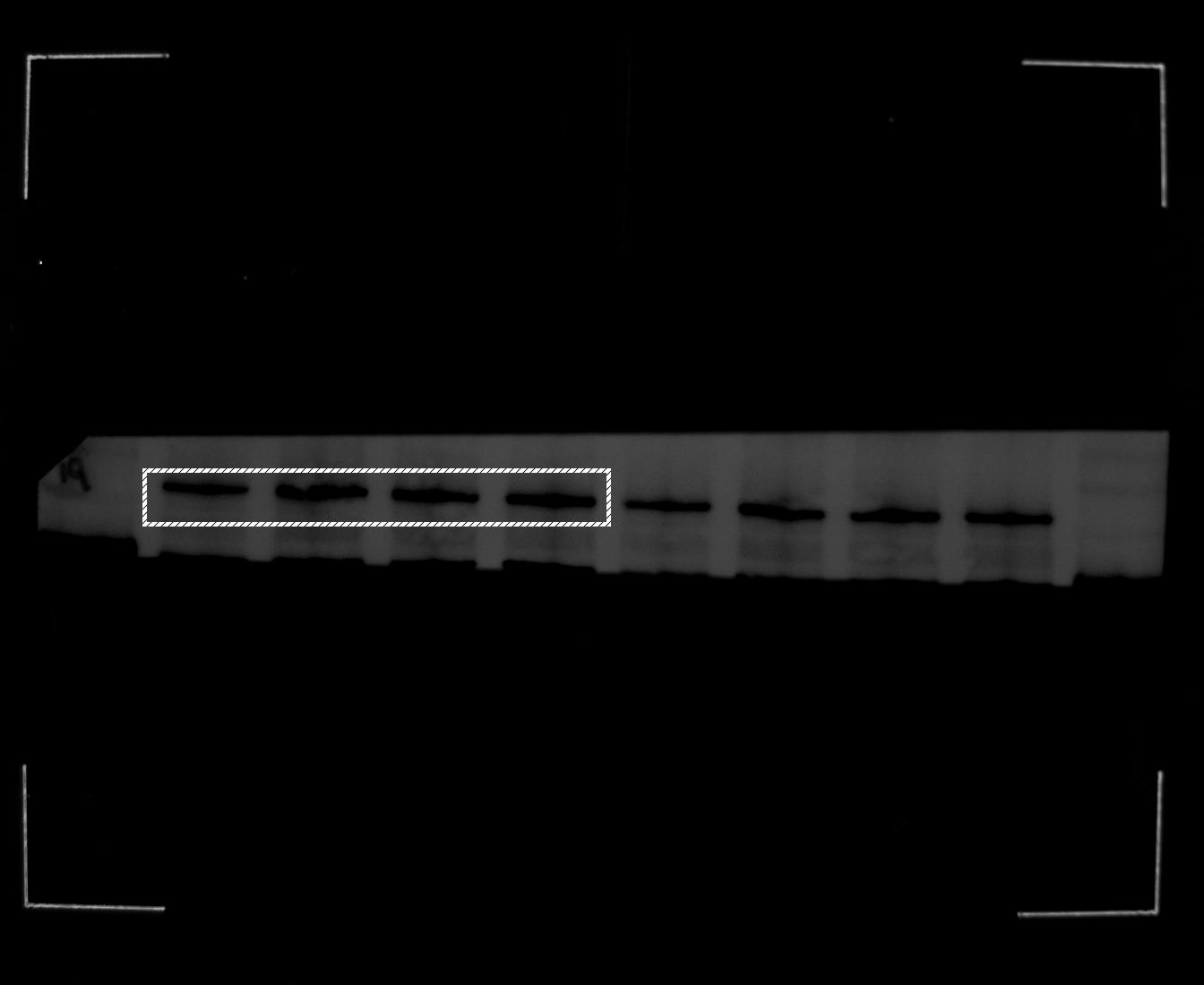
**

80kd

**TUBULIN**

**
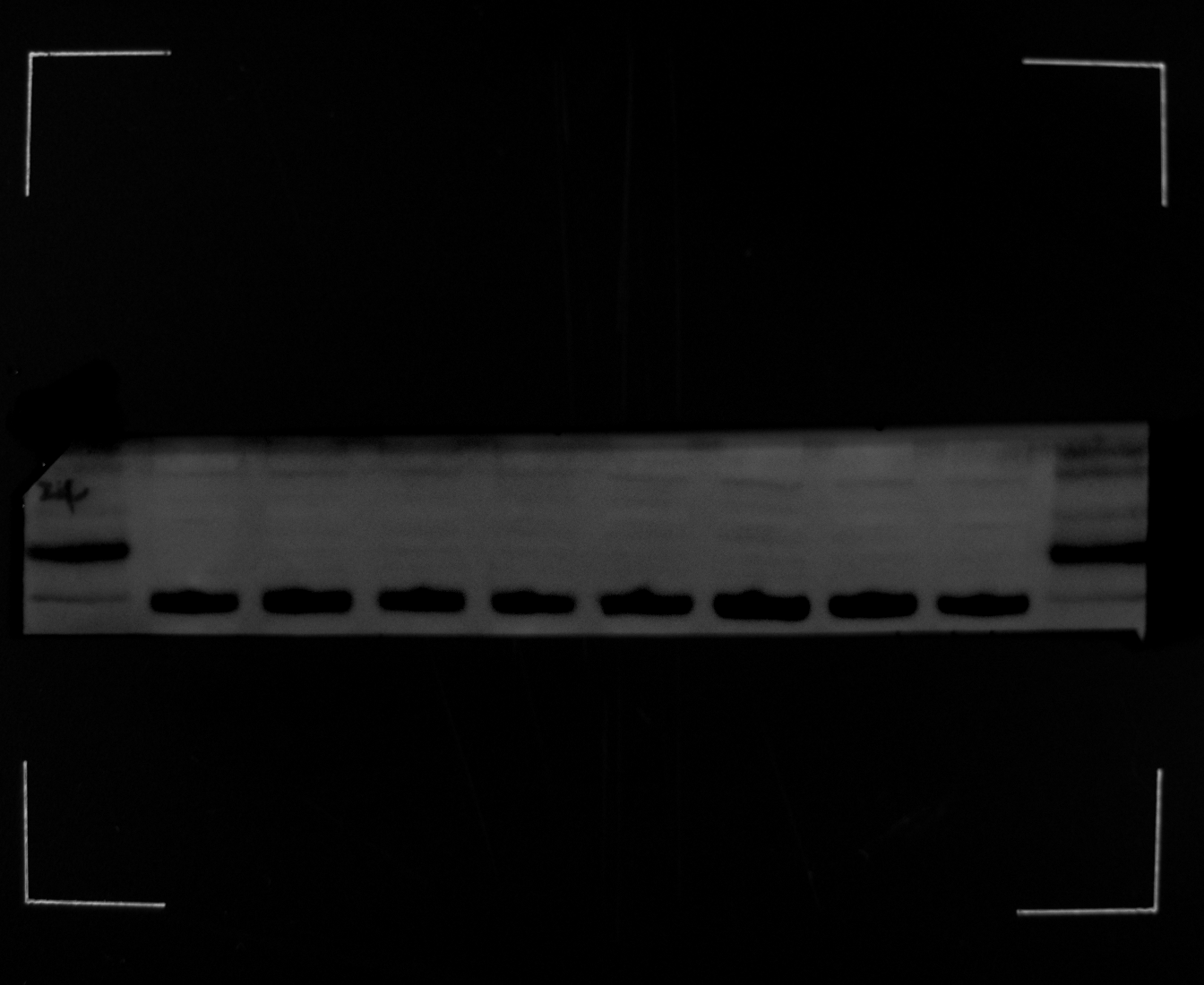
**

**C O N I**

55kd

**ARG-1**

**
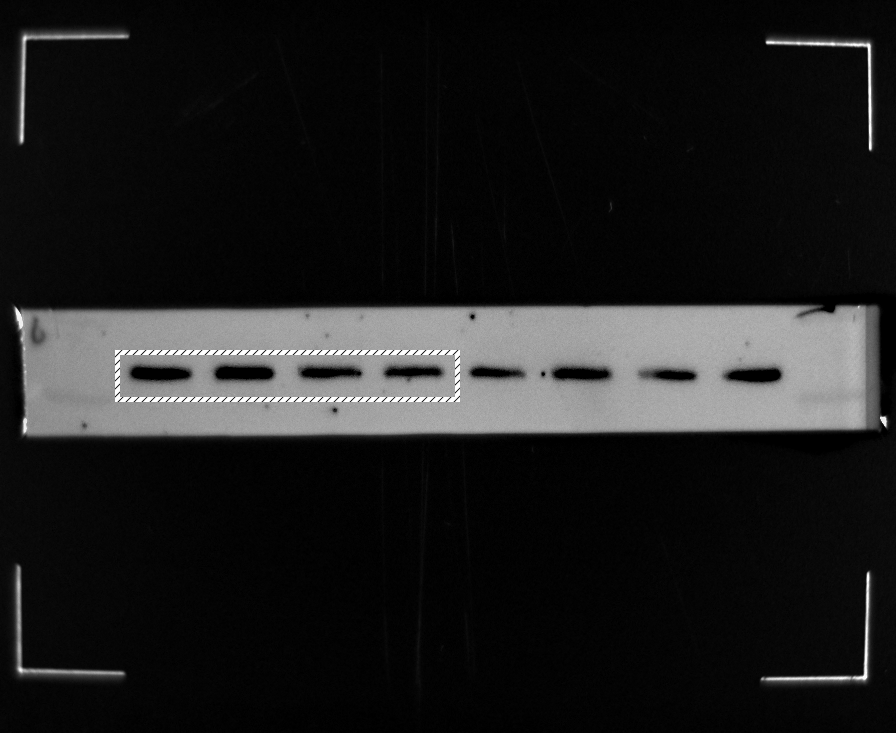
**

**C O N I**

35kd

**IBA1**

**
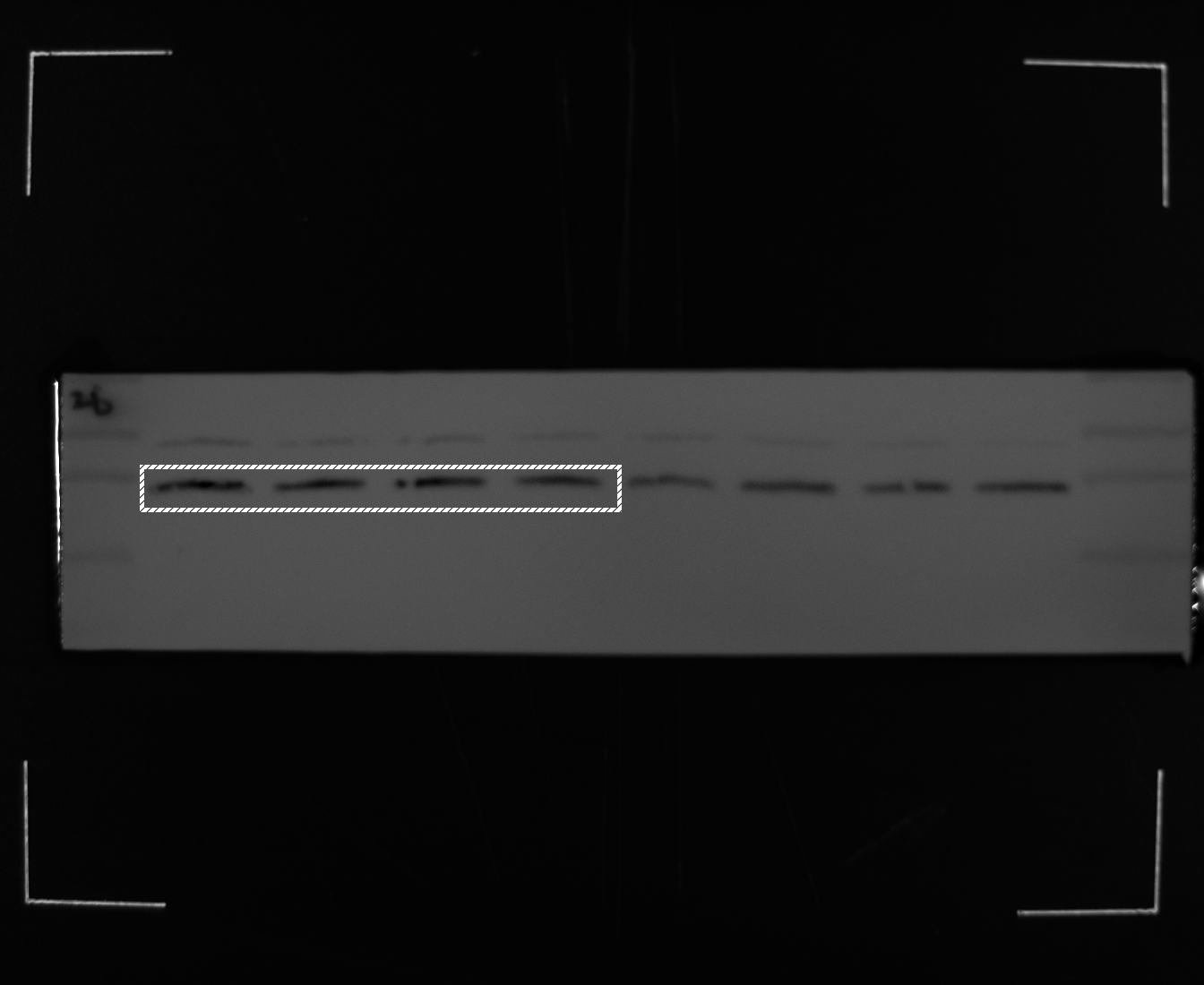
**

**C O N I**

17kd
