# Supplementary material for: The critical role of matrix metalloproteinase 9-mediated microglial polarization in perioperative neurocognitive disorders of aged rats
Source: Front Immunol. 2025 Aug 21;16:1650254. doi: 10.3389/fimmu.2025.1650254 (PMC12408272; doi:10.3389/fimmu.2025.1650254)
Supplement: Supplementary file 2 [file Table2.docx]

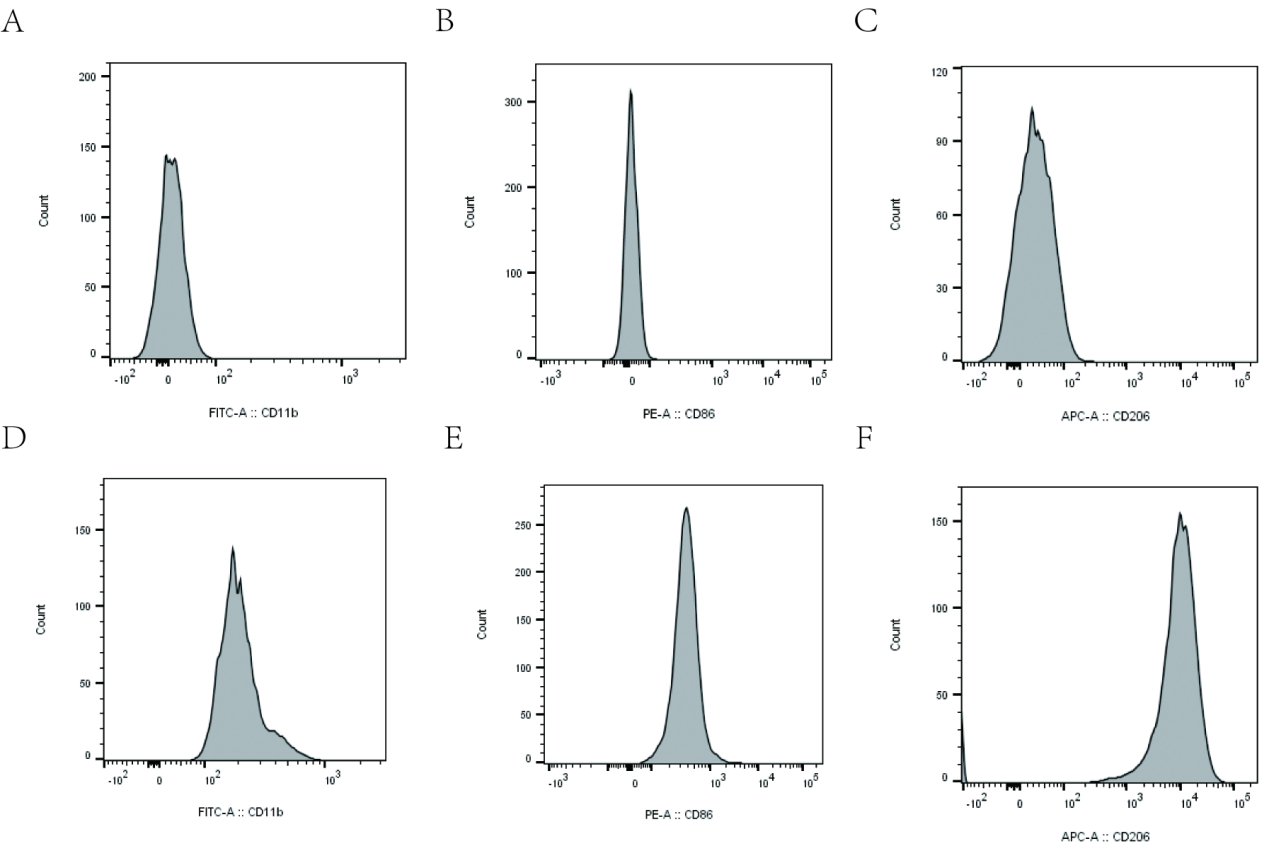


**FIGURE S2** Flow cytometry of the rat hippocampus unstained control and representative single-stained samples. **(A)** Fluorescence intensity of representative CD11b in unstained samples. **(B)** Fluorescence intensity of representative CD86 in unstained samples. **(C)** Fluorescence intensity of representative CD206 in unstained samples. **(D)** Fluorescence intensity of CD11b in representative single-stained samples. **(E)** Fluorescence intensity of CD86 in representative single-stained samples. **(F)** Fluorescence intensity of CD206 in representative single-stained samples.
